# Development of organophosphate hydrolase activity in a bacterial homolog of human cholinesterase

**DOI:** 10.3389/fchem.2014.00046

**Published:** 2014-07-16

**Authors:** Patricia M. Legler, Susanne M. Boisvert, Jaimee R. Compton, Charles B. Millard

**Affiliations:** ^1^Naval Research Laboratory, Center for Bio/Molecular Science and EngineeringWashington, DC, USA; ^2^Department of Chemistry, Texas State UniversitySan Marcos, TX, USA; ^3^Nova Research Inc.Alexandria, VA, USA; ^4^United States Army Medical Research and Materiel CommandFort Detrick, MD, USA

**Keywords:** organophosphate, cholinesterase, directed evolution, catalytic bioscavenger, nerve agent, hysteresis

## Abstract

We applied a combination of rational design and directed evolution (DE) to *Bacillus subtilis* p-nitrobenzyl esterase (pNBE) with the goal of enhancing organophosphorus acid anhydride hydrolase (OPAAH) activity. DE started with a designed variant, pNBE A107H, carrying a histidine homologous with human butyrylcholinesterase G117H to find complementary mutations that further enhance its OPAAH activity. Five sites were selected (G105, G106, A107, A190, and A400) within a 6.7 Å radius of the nucleophilic serine Oγ. All 95 variants were screened for esterase activity with a set of five substrates: pNP-acetate, pNP-butyrate, acetylthiocholine, butyrylthiocholine, or benzoylthiocholine. A microscale assay for OPAAH activity was developed for screening DE libraries. Reductions in esterase activity were generally concomitant with enhancements in OPAAH activity. One variant, A107K, showed an unexpected 7-fold increase in its *k*_cat_/*K*_m_ for benzoylthiocholine, demonstrating that it is also possible to enhance the cholinesterase activity of pNBE. Moreover, DE resulted in at least three variants with modestly enhanced OPAAH activity compared to wild type pNBE. A107H/A190C showed a 50-fold increase in paraoxonase activity and underwent a slow time- and temperature-dependent change affecting the hydrolysis of OPAA and ester substrates. Structural analysis suggests that pNBE may represent a precursor leading to human cholinesterase and carboxylesterase 1 through extension of two vestigial specificity loops; a preliminary attempt to transfer the Ω-loop of BChE into pNBE is described. Unlike butyrylcholinesterase and pNBE, introducing a G143H mutation (equivalent to G117H) did not confer detectable OP hydrolase activity on human carboxylesterase 1 (hCE1). We discuss the use of pNBE as a surrogate scaffold for the mammalian esterases, and the importance of the oxyanion-hole residues for enhancing the OPAAH activity of selected serine hydrolases.

## Introduction

**Figure 1:**
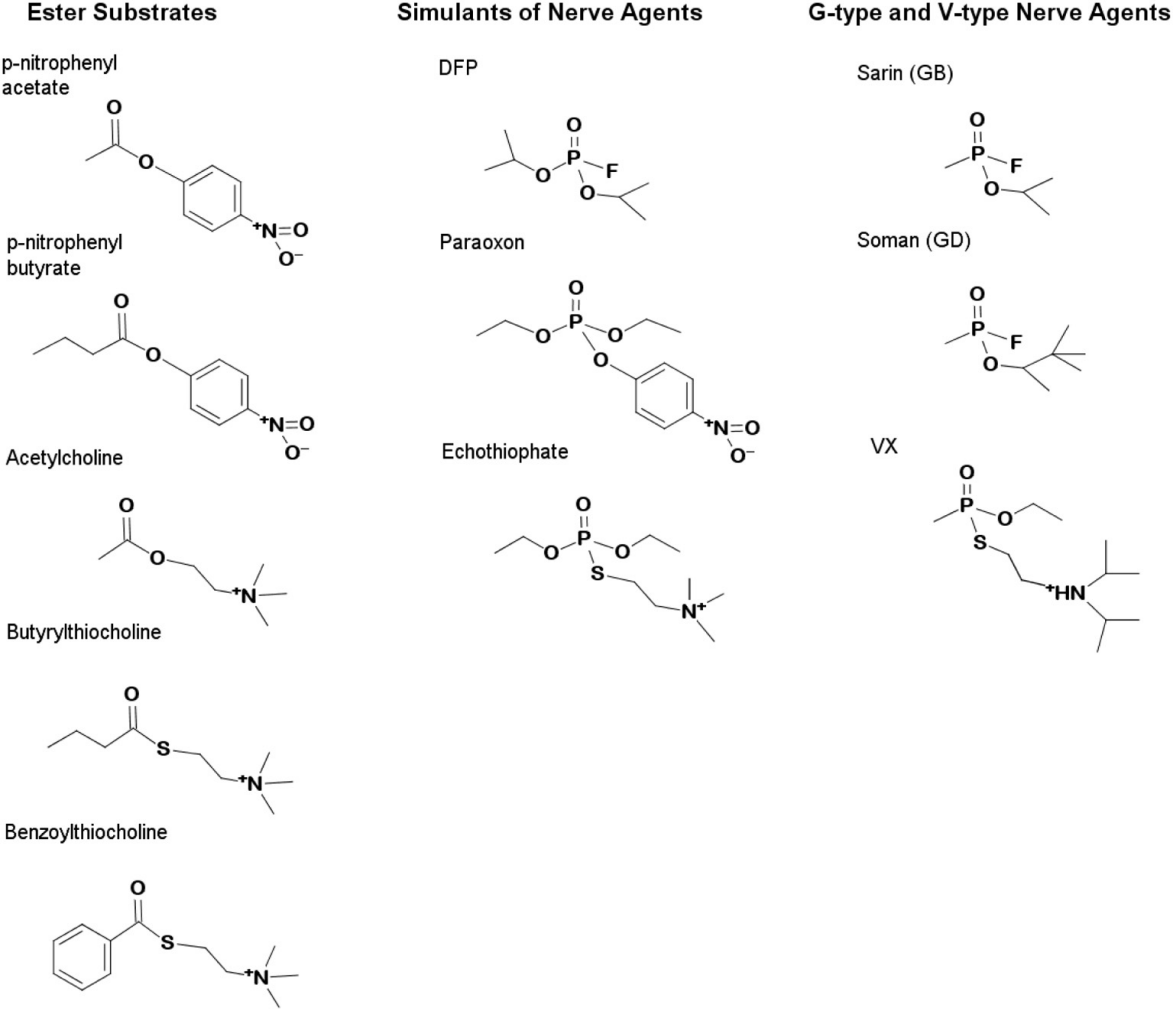
**Structures of carboxylester substrates and organophosphate inhibitors**. The G-type agents, Soman and Sarin, carry neutral R-groups while the V-type inhibitors, VX and echothiophate, contain cationic R-groups which mimic choline. Simulants which carry poorer leaving groups are commonly used in screening and include paraoxon, DFP, and echothiophate. OP are effective inhibitors because they mimic the substrates of the esterases which they inhibit. The transition states of carboxylesters are tetrahedral, while those of OP are pentavalent. Accommodation of the various R-groups of the OP is therefore determined empirically using a series of inhibitors with R-groups varying in size or charge.

Butyrylcholinesterase (BChE; EC 3.1.1.8) and its genetically engineered variants are being developed as therapeutic enzyme “bioscavengers” of organophosphorus acid anhydrides (OPAA) to prevent or treat OPAA poisoning (Millard et al., 1995a; Doctor and Saxena, 2005; Saxena et al., 2006) and also have been investigated to reverse cocaine addiction (Xie et al., 1999; Zheng and Zhan, 2008; Masson and Rochu, 2009). OPAA compounds (Figure 1) are highly toxic or lethal primarily because they rapidly, completely, and irreversibly inhibit essential biological stores of synaptic acetylcholinesterase (AChE; EC 3.1.1.7) leading to rigid paralysis, asphyxiation, and seizures (Shih et al., 2003). OPAA are archetypical irreversible inhibitors of serine hydrolases (Scheme S1), but in some cases the inhibition is slowly reversed (half-time of hours or days) because the phosphylated esterase undergoes spontaneous hydrolysis of the covalent adduct to yield reactivated enzyme (Main, 1979). Human BChE has been proposed as a prophylactic antidote because it is able to react rapidly with essentially all toxic pesticides and military “nerve agents” in the blood stream to prevent inhibition of AChE (reviewed in Ashani, 2000; Doctor and Saxena, 2005; Nachon et al., 2013).

The primary limitation to employing natural human BChE as a therapeutic is that each enzyme molecule can react only once with an OPAA inhibitor molecule and therefore will require an estimated dose of 200–1820 mg/70 kg of BChE to confer protection against 2 × LD_50_ of most nerve agents (Ashani, 2000; Geyer et al., 2010). For therapeutic enzyme bioscavengers, catalyzed turnover could significantly enhance the rate of OPAA hydrolysis and reduce the amount of enzyme needed for protection. Using rational protein design, Millard and colleagues introduced a single histidine residue (G117H) into the oxyanion hole of human BChE to increase the rate of spontaneous reactivation and thereby convert OPAAs from inhibitors into xenobiotic substrates which could be hydrolyzed by the mutant enzyme (Millard et al., 1995a; Lockridge et al., 1997). G117H enhanced the hydrolysis of paraoxon or echothiophate by 100,000-fold (Lockridge et al., 1997), and a second mutation (G117H/E197Q) permitted hydrolysis of even the most toxic nerve agents known (soman, sarin, or VX) by increasing the rate of spontaneous reactivation and simultaneously decreasing an unwanted side reaction known as “aging” (Scheme S1) (Shafferman et al., 1996; Millard et al., 1998).

**Figure 2:**
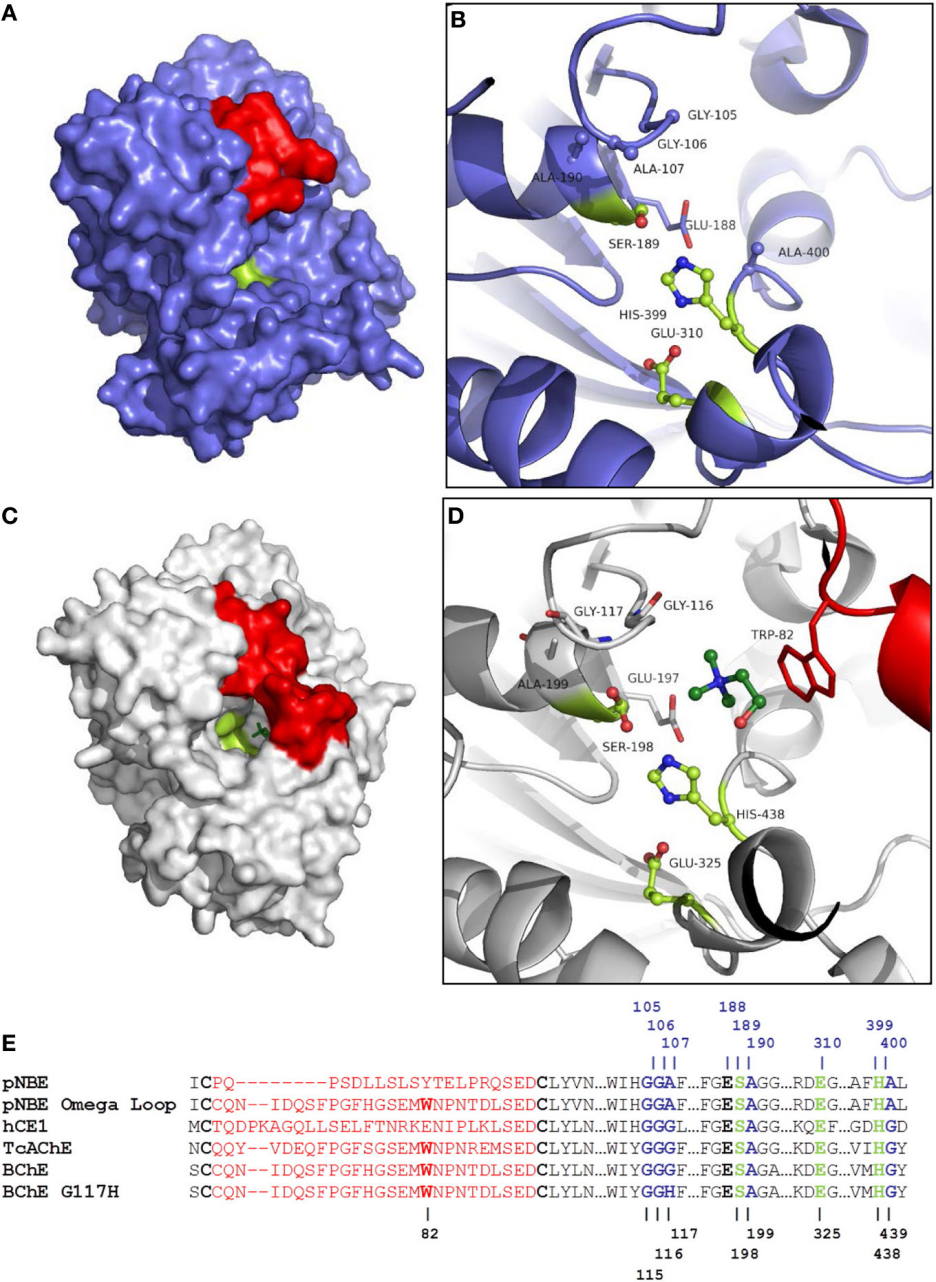
**Comparison of pNBE and BChE**. **(A)** Structure of pNBE (PDB 1QE3) (Spiller et al., 1999). **(B)** Active site of WT pNBE. The catalytic triad, Glu-310, His-399, Ser-189, is shown in lime. The residues selected for DE (G105, G106, A107, A190, and A400) are shown in blue ball and stick representation. The A107 residue is equivalent to G117 in butyrylcholinesterase. Structured residues between Cys-61 and Cys-82 corresponding to the Ω-loop of BChE are shown in red. pNBE and BChE are structurally similar and two structures can be superposed with an rmsd = 2.1 Å over 350 C_α_. **(C)** Structure of BChE (PDB 1P0M) (Nicolet et al., 2003). The Ω-loop of BChE is shown in red, choline is shown in dark green. The narrow gorge of BChE is partially formed by the Ω-loop. The catalytic triad is found at the bottom of the gorge. **(D)** The Ω-loop forms part of the choline binding site and carries Trp-82; this residue forms an energetically significant cation-pi interaction with cationic choline substrates (Ordentlich et al., 1993, 1995). Glu-197 also plays an important role in choline binding (Ordentlich et al., 1995; Masson et al., 1997b), and a residue equivalent to Glu-197 is present in pNBE. **(E)** Partial sequence alignment of pNBE, the pNBE Ω-loop variant, hCE1, TcAChE, BChE, and BChE G117H variant. The Ω-loop residues between Cys-65 and Cys-92 are shown in red and are unstructured in pNBE [PDB 1QE3 (Spiller et al., 1999)]. The Ω-loop of BChE was transferred to pNBE to form the chimeric variant. The Ω-loop is well formed in hCE1, AChE, and BChE. The Trp residue of the choline binding site is notably absent from pNBE and hCE1. The roles of these residues in catalysis are shown in Figure S1.

Cholinesterase “aging” is an irreversible dealkylation of the phosphylated serine that proceeds through enzyme-catalyzed formation of a carbocation leaving group (Scheme S1) (Michel et al., 1967; Li et al., 2007; Masson et al., 2010). Dealkylation results in an anionic phosphoester adduct that is resistant to nucleophilic attack. Aging involves the same cholinesterase residues that stabilize the binding of positively charged leaving groups of choline esters or V-type nerve agents (VX and VR), including, Glu-197, and Trp-82 within the Ω-loop of BChE (Figure S1, Figure 2) (Hosea et al., 1996; Masson et al., 1997a; Kua et al., 2003). Cholinesterases are predominantly found in higher eukaryotes and the Ω-loop may have arisen specifically to bind and hydrolyze choline esters (Figure 2) because very few esterases react efficiently with cationic ligands (Cousin et al., 1996). Structurally related esterases [such as human carboxylesterase (hCE)] that lack the homologous Trp do not exhibit significant cholinesterase activity and do not undergo comparable aging after OPAA inhibition (Hemmert et al., 2010).

Human BChE and its variants offer several important advantages as therapeutic enzymes (Doctor and Saxena, 2005), and transgenic animals bearing the G117H BChE variant have shown limited resistance to OPAA poisoning (Wang et al., 2004). A pegylated WT BChE enzyme (Protexia®) has also shown protection *in vivo* against soman and VX (Lenz et al., 2007; Mumford and Troyer, 2011). In addition to BChE, other enzymes such as AChE, hCE, or the metalloenzyme paraoxonase (PON1) have shown promise as bioscavengers. Both BChE (Saxena et al., 2006; Lenz et al., 2007; Mumford and Troyer, 2011) and PON1 (Costa et al., 1990; Li et al., 1995; Valiyaveettil et al., 2011) have shown limited protection against nerve agent and OP-pesticide intoxication in animal models. PON1 has been mutated to hydrolyze both G-type (soman and sarin) and V-type (VX) nerve agents (Cherny et al., 2013; Kirby et al., 2013). While PON1 is able to hydrolyze selected OP nerve agents at much faster rates *in vitro* than G117H or hCE, the *K*_m_ values for WT PON1 and its variants are in the millimolar range (Otto et al., 2010). High turnover numbers can be achieved by PON1 at saturating concentrations of OPAA (Kirby et al., 2013) but these concentrations are well above the levels of nerve agent that can be tolerated in living systems (LD^soman^_50_ = 113 μg/kg = 0.00062 mmol/kg in mice; Maxwell and Koplovitz, 1990) and the IC_50_ of AChE (IC^soman^_50_ = 0.88–2.53 nM, IC^sarin^_50_ = 3.27–6.15 nM; Fawcett et al., 2009). Consequently, each class of enzyme bioscavenger has advantages and disadvantages (Trovaslet-Leroy et al., 2011), and efforts to improve binding and expand the substrate specificities of several candidates is ongoing (Otto et al., 2010; Trovaslet-Leroy et al., 2011; Kirby et al., 2013; Mata et al., 2014).

Unfortunately, the modest OPAA rate enhancements conferred on BChE by the G117H mutation have not been improved upon for the past two decades (Millard et al., 1995a, 1998; Lockridge et al., 1997). Emerging technologies for protein engineering, especially directed evolution (DE) or biological incorporation of unnatural amino acids into the active site to improve OPAAH rates, have not been applied to cholinesterases largely because these eukaryotic enzymes have complex tertiary structures with extensive post-/co-translational modifications (e.g., glycosylation, GPI-anchor, disulfides) and, therefore, are not amenable to facile manipulation and expression in prokaryotic systems (Masson et al., 1992; Ilyushin et al., 2013). In contrast, DE has been successfully applied to paraoxonase using variants of human PON1 which produce soluble and active enzyme in *E. coli* (Aharoni et al., 2004).

To explore a combination of rational design and DE methods on a bacterial enzyme that shares the cholinesterase fold, we selected *Bacillus subtilis* p-nitrobenzyl esterase (pNBE, EC 3.1.1.-; Spiller et al., 1999). We chose pNBE as a surrogate scaffold because: (i) the X-ray structures suggest that pNBE may represent a prokaryotic structural precursor to the cholinesterases (AChE or BChE) (Spiller et al., 1999), as well as to the related family of hCE (Figure S1); (ii) pNBE appears to have a more open active site (Figure 2) and was shown previously to permit DE modifications of substrate specificity loops without compromising protein folding (Giver et al., 1998; Spiller et al., 1999); and (iii) pNBE, like the family of hCE (Fleming et al., 2007), lacks the amino acid present in BChE and AChE that is known to promote the deleterious aging reaction (e.g., W82 of BChE) (Masson et al., 1997a). We created and screened a library of 162 pNBE variants to identify mutations which could enhance OPAAH activity and expand the substrate and inhibitor specificities of this enzyme. The mutations were then transferred to hCE1 to determine if pNBE could be used as a surrogate scaffold. We identified one pNBE variant with a three-order of magnitude enhancement in somanase activity compared with WT. Unexpectedly, the variant with the largest enhancement in OPAA activity also underwent a slow time- and temperature-dependent change in activity. We correlate our results with the solved X-ray structures of pNBE to understand possible mechanisms for engineered OPAAH activity, and discuss complications posed by hysteretic forms in the kinetic and structural analysis of mutant pNBE, AChE and BChE (Masson et al., 2005; Badiou et al., 2008; Lushchekina et al., 2014).

## Materials and methods

### Materials

BugBuster™ and the pTriEx-3 vector were from Novagen (San Diego, CA). Chelating Sepharose, Q-Sepharose, and PD-10 columns were from GE Healthcare Life Sciences (Piscataway, NJ). QuikChange™ kits were purchased from Stratagene (La Jolla, CA). Benzoylthiocholine (BzCh) was purchased from TCI America (Portland, OR). The 96-well, clear polystyrene HIS-Select® High Capactiy (HC) Nickel Coated Plates were purchased from Sigma. All other chemicals were purchased from Sigma. Echothiophate was from Wyeth Pharmaceuticals Inc. (Philadelphia, PA).

### Construction of the DE library

Five sites in pNBE were selected for the directed-evolution library: G105, G106, A107, A190, and A400. The C_α_ of each of the five residues was between 5.0 and 6.7 Å from the Ser-189-Oγ. The A107H mutation was also present in each starting variant with the exception of the twenty variants of A107. The plasmids of the DE library were synthesized by GeneArt, Inc. (Regensberg, Germany). The pNBE expression vector (pTriEx-3, Novagen Inc.) contained an N-terminal PreScission™ Protease cleavage site and a hexa-histidine tag preceding the pNBE sequence.

### Construction of the chimeric BChE/pNBE Ω-loop variant

The megaprimer method (Sarkar and Sommer, 1990) was used to construct the chimeric BChE-pNBE variant. A megaprimer containing a sequence from one of the known cholinesterase substrate specificity loops, in this case the “Ω-loop” of BChE, was used to replace the homologous sequence in pNBE. The variant was sequenced to confirm the substitution. Protein sequences are included in the Supplemental Information.

### Small scale protein expression and purification

All 95 constructs in the DE library expressed soluble protein under these conditions. Four milliliter cultures of LB containing 100 μg/mL Ampicillin were inoculated with frozen glycerol stocks and grown for 3 h at 37°C with shaking (200 rpm). Cultures were induced with 1 μL of 1.0 M IPTG overnight at 17°C. Bacteria were pelleted and then lysed in 0.5 mL Lysis Buffer (87.5% BugBuster™, 2 mM BME, 50 mM Tris pH 7.6, 375 mM NaCl) at room temperature (17–22°C) for at least 1 h. Lysed bacteria were centrifuged at 4800 × g for 10 min. Clarified lysates were then loaded onto nickel-charged Chelating Sepharose columns (0.5 mL slurry per disposable column) equilibrated with three column volumes of equilibration buffer (EB; 50 mM Tris pH 7.6, 500 mM NaCl, 2 mM BME). After the supernatant was loaded, the columns were washed again with three column volumes of EB. To elute contaminants, the columns were washed with three column volumes of EB containing 60 mM Imidazole. Proteins were isocratically eluted with EB containing 300 mM Imidazole. Imidazole readily reacts with the carboxyl ester substrates used to assay the enzyme; thus, it was necessary to buffer exchange the enzymes with BioMax (10,000 NMWL) ultrafiltration units three times with 50 mM HEPES pH 7.0, 150 mM NaCl to remove the imidazole. Purified enzymes ran as single bands in SDS-PAGE gels and were judged to be ≥90% pure.

### Large scale protein expression and purification

Large scale preps of selected variants were used for kinetic analysis. LB (1–3 L) containing 100 μg/mL Ampicillin was grown with shaking at 37°C and induced overnight with 0.2 mM IPTG at 17°C. Bacterial pellets were lysed in 40 mL of lysis buffer containing ~30 mg lysozyme and then sonicated for 1 min in an ice bath. Lysates were clarified by centrifugation (30 min at 20,500 × g). Supernatants were loaded onto a 20 mL nickel-charged Chelating Sepharose column. After loading, the column was washed with EB containing 60 mM imidazole until the A_280_ returned to a level baseline. Protein was eluted with EB containing 300 mM imidazole. Fractions containing pNBE were combined and dialyzed against 50 mM Tris pH 7.6, 150 mM NaCl, 2 mM BME. Protein was loaded onto a 30 mL Q-Sepharose column and eluted between 260 and 400 mM NaCl during the gradient.

### Carboxylesterase assays

Steady state kinetic parameters for the enzyme catalyzed hydrolysis of p-nitrophenyl acetate (pNPA) and p-nitrophenyl butyrate (pNPB) were measured in triplicate at room temperature in 50 mM HEPES 7.0, 150 mM NaCl (405 nm). Substrate and inhibitors were dissolved in DMSO and accounted for less than 1% of the reaction volume.

Acetylthiocholine (AtCh), butyrylthiocholine (BtCh), or benzoylthiocholine (BzCh) hydrolysis was measured in triplicate at 412 nm in cuvettes or a plate reader using Ellman's reagent (0.5 mM DTNB) (Ellman et al., 1961). All assays were done in 1× Sorensen's buffer (53.4 mM Na_2_HPO_4_, 13.4 mM KH_2_PO_4_) pH 7.4 at room temperature (22 ± 2°C). An extinction coefficient of 13.6 mM^−1^cm^−1^ was used for calculations. One Unit of activity (U) was defined as 1 μmol product produced per min, and specific activity (S.A.) was defined as Units per milligram of enzyme (U/mg).

### Primary assay for screening

HIS-Select® plates were washed once with 200 μL of binding buffer (50 mM Hepes pH 7.0, 150 mM NaCl). Each his-tagged protein (~25 mU) in the same buffer (100 μL) was added to two wells and allowed to bind for 1 h at 37°C. All wells contained enzyme after each plate setup. The OPAA inhibitor was added (0.5–5 μL) to one of the two wells and incubated for 10 min at room temperature. Cautionary note: the OPAA compounds used in this study are highly toxic and must only be handled with adequate legal authority, training, and safety precautions. Liquid was removed by a multichannel pipettor, and plates were washed four times with 200 μL of appropriate reaction buffer. Buffer (90 or 95 μL) and 0.5 M EDTA (10 or 5 μL) were then added to each well to elute the protein. Plates were left at room temperature or at 37°C, and aliquots of enzyme (10 μL) were removed over time and assayed in separate 96-well plates using 5 mM pNP-butyrate in binding buffer. Activity was measured at 4–6 time points to confirm reactivation of a single clone. For the clones which reactivated in the 96-well assay, large scale preps were then used to more accurately quantitate the enhancements in the rates of reactivation.

### Large scale discontinuous spontaneous reactivation assays

Spontaneous reactivation was measured essentially as previously described (Millard et al., 1995a; Lockridge et al., 1997). Briefly, an aliquot of uninhibited enzyme or the OPAA-inhibited (>95% inhibited) enzyme was loaded onto PD-10 gel filtration columns equilibrated with 50 mM Tris pH 7.6, 150 mM NaCl, 2 mM BME. At time *t* = 0, the columns were loaded, and the protein was rapidly eluted; fractions were incubated at 37°C, activity was measured for the uninhibited enzyme, and inhibited enzyme and percentages of reactivated enzyme were calculated. The pseudo first order rate constant for spontaneous reactivation due to the hydrolysis of the serinyl-phosphate adduct, *k*_r_, was determined by fitting the data to the following equation (Wang and Braid, 1967; Main, 1979):

$$A_{t}=A_{\max}\text{ ​}\left(1-e^{-k_{r}t}\right)$$

where *A_t_* is the percent reactivated at time *t* and *A*_max_ is the maximal percent reactivated at final observation time >> *t*_0_. For the A107H/A190C variant, which exhibited a form of hysteresis (Hanozet et al., 1981; Uto and Brewer, 2008), the enzyme was incubated at 37°C for at least 2 h after exchanging the buffer using a PD-10 column equilibrated with 50 mM Tris pH 7.6, 150 mM NaCl, 2 mM BME. The enzyme was then inhibited, and rates of reactivation were measured.

### Organophosphate inactivation

Aliquots of enzyme were inhibited with different concentrations of inhibitor, and the activity was measured discontinuously using pNP-butyrate at different time points. Data were plotted and fit to a single exponential decay equation to obtain *k_obs_*, the observed first order rate constant. A secondary plot was used to determine the maximal rate constant for inactivation, *k*_2_, at infinite inhibitor concentration. The rate constant was determined by plotting *k_obs_* vs. [*I*] concentration and fitting the data to the following equation (or by extrapolation using the double-reciprocal form of the equation) from Kitz and Wilson (1962):

$$k_{obs}=\frac{k_{2}}{1+K_{p}/[I]}$$

The apparent bimolecular rate constant, *k_i_*, for formation of the covalent E-I complex from free enzyme and free inhibitor was calculated according to the following:

$$k_{i}=k_{2}/K_{p}$$

where *K_p_* is a Michaelis-type constant for the inhibitor.

## Results

### Selection of residues for directed evolution (DE)

Prior to the creation of the DE library, we produced the A107H pNBE variant by analogy with BChE G117H (Millard et al., 1995a; Lockridge et al., 1997) and demonstrated that it possesses increased OPAAH activity (Table 1). The OPAAH activity of the pNBE A107H variant was found to be acid-catalyzed and 4-fold higher at pH 7.0 than at pH 7.6 (Table 1). At pH 7.0 the reactivation rate of the A107H variant was 46-fold higher when compared with WT and 18-fold higher at pH 7.6.

**Table 1:** **pH dependence of reactivation rates after inhibition with ethyl paraoxon**.

**Enzyme**	**Inhibitor**	**pH**	**% Reactivation**	***k*_reactivation_ (1/h)**
WT	Paraoxon	7.6	110 ± 10%	0.03 ± 0.01
	Paraoxon	7.0	91 ± 8	0.05 ± 0.01
	Paraoxon	6.5	88 ± 6	0.035 ± 0.007
	Paraoxon	6.0	52 ± 2	0.042 ± 0.005
A107H	Paraoxon	7.6	102 ± 5	0.53 ± 0.09
	Paraoxon	7.0	90 ± 10	2.3 ± 0.3
BChE Ω Loop Mutant with A107H	Paraoxon	7.6	86 ± 4	1.0 ± 0.1

Rates were measured in 50 mM Tris pH 7.6, 150 mM NaCl, 2mM BME; 50 mM Hepes 7.0, 150 mM NaCl, 2 mM BME; 50 mM MES pH 6.5, 150 mM NaCl, 2 mM BME; or 50 mM MES pH 6.0, 150 mM NaCl, 2 mM BME at 37°C.

To identify mutations which could further enhance the OPAAH activity of A107H, we constructed a DE library of double mutants at five different sites: A107H/G105X, A107H/G106X, A107H/A190X, and A107H/A400X (where X stands for any amino acid). We also examined the A107X single mutation variants of pNBE. Each residue selected for DE (G105, G106, A107, A190, or A400) was within 6.7 Å of the Oγ of the nucleophilic Ser-189 in pNBE and was conserved in BChE and hCE1 (Figure 2). Based upon the X-ray structure of pNBE, we concluded that the backbone NH groups of G106, A107, and A190 form a 3-point oxyanion hole (Figure S1). Gly-105 is situated near the oxyanion hole, but is not part of the oxyanion hole. The corresponding G105A variant in human AChE affected the turnover number of the substrate, but not the *K*_m_; this substitution was suggested to affect the conformational mobility of the adjacent residues of the oxyanion hole (Ordentlich et al., 1998). The side-chain of Ala-190 was hypothesized to exert an effect on the polarity and/or orientation of the backbone NH groups of A107 and G106 and thereby affect TS stabilization. The oxyanion hole is the primary source of transition state stabilization in serine hydrolases (Bryan et al., 1986). The A190 side-chain is situated directly behind the loop carrying A107 and G106. The C_β_ of A190 is 3.6–3.7 Å away from the backbone NH of A107 and G106 (Figures S1B,D). The A400 residue is located on a loop of pNBE. The A400T mutation in pNBE was shown previously to project into the active site (6.7 Å from the Ser-189-Oγ) and enhance the thermostability of pNBE in DE experiments by Spiller et al. (1999). Spiller et al. proposed that the Thr side-chain of residue-400 may stabilize His-399 of the catalytic triad. A400 was also near the choline leaving group in overlays of pNBE with a BChE-choline co-crystal structure (1P0M) (Nicolet et al., 2003) (Figure S1C). We selected it here to find variants which might stabilize a particular conformer of His-399 or stabilize the alkyl groups of the soman pinacolyl group, the DFP *iso*-propyl groups, or, alternatively, the cationic choline-like leaving groups of V-type nerve agents and simulants (e.g., echothiophate).

### Substrate specificity

Five substrates were tested with single point assays and the DE library of variants to determine if the mutations altered substrate specificities: pNPA, pNPB, AtCh, BtCh, and BzCh (Figure S2). WT pNBE had the highest substrate specificity for pNP-butyrate as judged by the bimolecular rate constant, *k*_cat_/*K*_m_ = 14,000 ± 2000 min^−1^ mM^−1^. A detectible level of CE activity is needed to measure reactivation rates by the discontinuous method. All 95 of the variants had detectable levels of CE activity when pNP-butyrate was used as the substrate. This allowed the use of a common substrate for activity measurements at different time points during reactivation experiments. No significant enhancement in the substrate specificities of the DE library variants for pNPA or pNPB was observed.

### Characterization of variants with enhanced cholinesterase activity

Ideally, universal OP bioscavenging enzymes should scavenge both G-type and V-type agents (Figure 1). V-type agents, such as VX and VR, and V-type simulants like echothiophate mimic positively charged choline esters (Scheme S1) and readily inhibit AChE and BChE. Echothiophate and VX are slowly turned over by the BChE G117H variant (Millard et al., 1995a). Cholinesterase activity can only be found in a subset of esterases, typically those of eukaryotes (Cousin et al., 1996). The cationic choline esters are accommodated by two key residues at the bottom of the gorge of BChE and AChE, Trp-84/82, and Glu-199/197 (TcAChE/BChE numbering) (Ordentlich et al., 1995). These residues also play a role in the binding specificity of tetrahedral cationic V-type agents in AChE (Hosea et al., 1996), as well as in the unfavorable “aging” process (Shafferman et al., 1996). A residue within the peripheral anionic site (PAS) at the top of the gorge, Asp-72/70, also plays a role in V-type agent binding (Hosea et al., 1996), but is relatively distant from the choline binding pocket (~7 Å); hCE1 and pNBE lack a homologous Asp residue (Figure 2E). Since hCE1 and pNBE are structurally similar to AChE and BChE (Figure S1A) but are not known to hydrolyze choline esters or become inhibited by V-type agents, we also examined the DE library for the development of cholinesterase activity and susceptibility to inhibition by echothiophate (last section).

Cholinesterases contain an omega-shaped loop between the disulfide bonded cysteines, Cys-67 and Cys-94 (*Tc*AChE numbering) (Figure 2, Figure S1). The Ω-loop carries Asp-72/70 and Trp-84/82 of the choline binding site. To determine if a cholinesterase Ω-loop could be inserted, we substituted the Ω-loop sequence of BChE into the pNBE A107H variant. The chimeric variant folded as a functional esterase (Table 2). The *K*_m_ and *k*_cat_ values for pNPA were similar to those of the WT enzyme. However, the loop insertion alone did not confer cholinesterase activity, and the *k*_cat_ and *K*_m_ for BzCh and BtCh were similar to those of the A107H pNBE variant (Table 3). Thus, the DE library was made with the A107H pNBE variant, rather than the loop-insertion variant.

**Table 2:** **Substrate specificities of pNBE and selected variants**.

**Enzyme**	**Substrate**	*****k***_**cat**_ (1/min)**	*****K***_**m**_ (mM)**	***k***_**cat**_/***K***_**m**_ (1/min*mM)
WT	pNPA	370 ± 30	1.2 ± 0.3	300 ± 80
	pNPB	1100 ± 40	0.08 ± 0.01	14000 ± 2000
A107H	pNPA	130 ± 10	5.6 ± 0.7	23 ± 3
	pNPB	520 ± 20	0.12 ± 0.02	4300 ± 700
A107H/A190C	pNPA	70 ± 10	0.9 ± 0.4	70 ± 30
	pNPB	7 ± 1	0.3 ± 0.1	20 ± 10
A107H/A400T	pNPB	460 ± 10	0.12 ± 0.02	3800 ± 600
A107H/A400V	pNPB	510 ± 30	0.17 ± 0.03	3000 ± 600
BChE Ω Loop Mutant with A107H	pNPA	185 ± 6	1.6 ± 0.1	116 ± 8

pNPA (pNP-acetate) and pNPB (pNP-butyrate) assays were run in 50 mM HEPES pH 7.0, 150 mM NaCl, 22 ± 3°C. All enzymes had the N-terminal His-tag.

**Table 3:** **Steady state kinetic parameters for selected pNBE variants of the DE library**.

**Substrate**	**Benzoylthiocholine^a^**			**Butyrylthiocholine^b^**		
**Enzyme**	***k***_**cat**_ **(1/min)**	***K***_**m**_ **(mM)**	***k***_**cat**_/***K***_**m**_(1/min*mM)	***k***_**cat**_ **(1/min)**	***K***_**m**_ **(mM)**	***k***_**cat**_/***K***_**m**_(1/min*mM)
WT	70 ± 9	1.2 ± 0.3	58 ± 16	130 ± 10	5.4 ± 0.8	24 ± 4
A107H	13 ± 1	0.6 ± 0.2	22 ± 7	35 ± 8	17 ± 5	2.0 ± 0.9
A107H Ω Loop	8 ± 1	0.9 ± 0.3	9 ± 3	10.4 ± 0.9	8.0 ± 0.7	1.3 ± 0.2
A107K	570 ± 50	1.4 ± 0.2	410 ± 70	>20	>8^c^	–
A107Q	40 ± 4	1.0 ± 0.2	39 ± 9	40 ± 10	19 ± 7	2 ± 1
A107R	90 ± 20	5 ± 1	20 ± 6	>50	>8^c^	–
A107S	39 ± 9	1.4 ± 0.6	30 ± 10	780 ± 30	14.4 ± 0.7	54 ± 3
A107T	36 ± 3	0.6 ± 0.2	60 ± 20	240 ± 30	11 ± 2	22 ± 5
A107V	38 ± 4	0.5 ± 0.2	80 ± 30	56 ± 8	8 ± 2	7 ± 2
A107Y	21 ± 2	0.6 ± 0.1	35 ± 8	45 ± 5	6.0 ± 0.9	7 ± 1
A107H/A190G	29 ± 4	0.9 ± 0.3	30 ± 10	50 ± 30	11 ± 7	5 ± 4
A107H/A190R	12 ± 1	0.6 ± 0.2	20 ± 7	200 ± 30	13 ± 2	15 ± 3
A107S/A190G	23 ± 4	2.2 ± 0.6	10 ± 3	90 ± 30	11 ± 4	9 ± 4
A107V/A190G	21 ± 2	0.6 ± 0.1	35 ± 7	45 ± 5	6.0 ± 0.9	8 ± 1
A107H/A400D	80 ± 10	2.1 ± 0.6	40 ± 10	190 ± 60	11 ± 5	18 ± 9
A107H/A190S/A400S	6.4 ± 0.9	0.8 ± 0.2	9 ± 3	115 ± 14	9 ± 1	13 ± 3

Benzoylthiocholine and butyrylthiocholine were used as substrates. Specific activities of the other variants are shown graphically in the Supplemental Information.

a Benzoylthiocholine has limited solubility in DMSO, the highest substrate concentration tested was 2.5 mM.

b Butyrylthiocholine was also a poor substrate of pNBE, and K_m_ values were in the mid-millimolar range. Saturation was not achieved at the highest substrate concentration tested (8 mM). K_m_ values were extrapolated from double reciprocal plots.

c Saturation was not achieved at [S] = 8 mM, and the plot of velocity vs. [S] was linear. Extrapolated K_m_'s exceeded 40 mM.

All 95 variants were initially examined for cholinesterase activity using single point assays (Figure S2). To determine if the pNB-esterase variants could bind and turnover cationic OPAA like echothiophate, we first looked for cholinesterase activity. AChE, BChE, hCE1, and pNB-esterase all share the same fold (Figure S1A). Steady state kinetic parameters for the variants which showed significant increases in cholinesterase activity are shown in Table 3. Unexpectedly, the variant which showed the largest increase in cholinesterase activity was a single mutant with a positively charged lysine residue, A107K. This variant showed a 7-fold increase in the *k*_cat_/*K*_m_ and an 8-fold increase in the *k*_cat_ of benzoylthiocholine, while the *K*_m_ was similar to WT. Substitution of Arg (A107R) in place of Lys did not significantly enhance benzoylthiocholinesterase activity, but resulted in a 3-fold higher *K*_m_ suggesting that the larger Arg side-chain may interfere with substrate binding. Substitution of A107 by the neutral residue, Gln, and by hydrophobic residues yielded similar *K*_m_ values and no enhancement of *k*_cat_. Substitution of A107 by His also did not confer significant cholinesterase activity.

Butyrylthiocholinesterase activity was the highest in the A107S, A107T, A107H/A190R, and A107H/A400D variants (Table 3). A400 was predicted to be near the choline group from structural overlays. The A107H/A400D variant had a 2-fold increase in the *k*_cat_/*K*_m_ for benzoylthiocholine and 9-fold increase for butyrylthiocholine when compared to A107H; however, the *K*_m_ values for all of the variants were >1 mM, indicating that the pNBE variants could only weakly bind cationic substrates.

### Optimization of the primary assay used for screening the DE library

**Figure 3:**
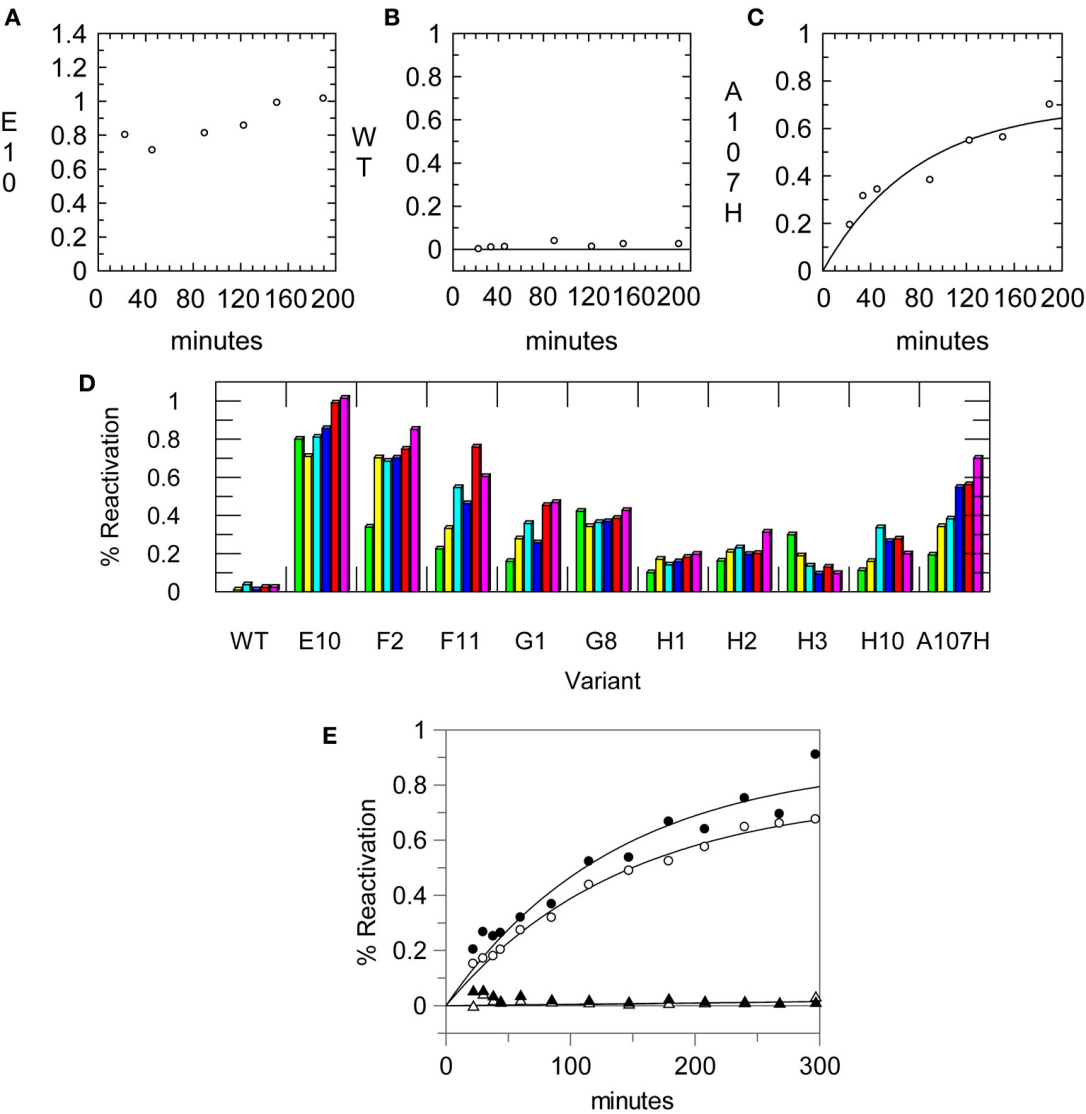
**Reactivation data from the primary assay using a 96-well His-Select® plate**. Aliquots of enzyme were removed once at each time point and assayed for CE activity using pNP-butyrate. Enzymes were reactivated in 50 mM Tris pH 7.6, 150 mM NaCl. Reactivation rates measured using the assay for **(A)** the A107H/A190C (E10) variant, **(B)** the WT pNBE, and **(C)** the A107H variant. **(D)** Example of reactivation rates using single point measurements for selected variants at different time points using the 96-well plate assay. Some variants showed full reactivation by the first time point while others progressively reactivated over longer time periods. **(E)** Reactivation rates measured for the A107H variant using the 96-well plate assay after one (∘) or two (•) washes to remove the inhibitor, Paraoxon. The reactivation of the WT enzyme is also shown after one (▴) or two (▵) washes for comparison. Rates were measured in 50 mM Tris pH 7.6, 150 mM NaCl at 37°C.

To develop a micro-scale assay for reactivation, (His)_6_-tagged enzymes were bound to nickel-coated 96-well plates. To maintain near physiological conditions, the pH was kept at 7.6; measurement at a sub-optimal pH also allowed for a longer time period to carry out the subsequent steps. Two wells were coated with enzyme (≤0.025 U per well) for each variant to measure the activity of the uninhibited and inhibited enzyme. The enzyme was inhibited on the plate, and excess enzyme and inhibitor were removed. The plates were then washed with buffer. Rates of reactivation were comparable after one, two, or four washes. For the plate assay, four washes were done to ensure removal of the OPAA. After washing away excess inhibitor and unbound enzyme, the enzyme was eluted from the plate with 50 mM EDTA. Imidazole was avoided because it readily reacted with the ester substrates (Bruice and Schmir, 1956). Aliquots were removed and assayed over time. The rate constant for reactivation for A107H using the microscale assay (*k*^2washes^_r_ = 0.22 ± 0.08 h^−1^; *k*^4washes^_r_ = 0.3 ± 0.2 h^−1^) was comparable with that determined using a gel filtration column (*k*_r_ = 0.53 ± 0.09 h^−1^) at the same pH and temperature. Data collected using the microscale assay and 2 washes are shown in Figure 3. The DE library was screened one to two times with the various OP. From the first round, 26 of the 95 variants were more carefully examined with large scale preps and kinetic experiments. Error in the values of *k*_r_ was higher using data collected from the microscale assay, suggesting that it is better suited for large-scale screening than for precise determination of kinetic parameters.

For slow and moderate rates of reactivation the microscale assay was useful as a primary assay for the exploration of OP inhibitors and reaction conditions (Figure 3D). The microscale assay helped identify the variants which could reactivate faster than the single variant, A107H. The vast majority of the variants did not show significant enhancements in OPAAH activity using either the discontinuous assay or a continuous assay with paraoxon; these results are consistent with other applications of DE (Dalby, 2003; Goldsmith and Tawfik, 2013). Using the OPAA activity of A107H as a screening threshold, approximately 3% of the library was advanced for further testing.

The half time of reactivation for pNBE A107H was *t*_1/2_ = 78 min. Thus, observation of full reactivation at ~20 min indicated that the *k*_r_ was ≥1.9 h^−1^ or ≥4-fold faster than the A107H variant. Reactivation rates for the top hits were more accurately measured using large scale preps of the enzymes and gel filtration columns.

It should be noted that the measurement of reactivation rates alone cannot identify a mechanism of OP resistance. Resistance to OP inhibition can arise from reduced binding of OP, poor stabilization of the TS, poor accommodation of the R-groups as the pentavalent TS forms, or increased OPAAH activity. These possibilities can only be distinguished by additional kinetic experiments. These methods are routinely used to characterize pesticide resistance mechanisms in insecticide-insensitive AChE variants (Newcomb et al., 1997; Temeyer et al., 2012; Zhang et al., 2012).

### Variants with enhanced OPAAH activity

After screening the library of 95 variants for reactivation after paraoxon inhibition, three variants were found to reactivate faster than the A107H variant: A107H/A400M (H2), A107H/A190G (F2), and A107H/A190C (E10). The A107H/A190C double variant was found to have the greatest rate enhancement. Relative to the WT pNBE (*k*^Paraoxon^_r_ = 0.03 ± 0.01 h^−1^), the rate constant for reactivation following paraoxon inhibition was 18-fold higher for the A107H variant (*k*^Paraoxon^_r_ = 0.53 ± 0.09 h^−1^), and 50-fold higher for the A107H/A190C double variant (*k*_r_ = 1.5 ± 0.2 h^−1^) (Table 4). Consistent with the aliesterase hypothesis (Oppenoorth and van Asperen, 1960), the turnover number for pNBE-catalyzed hydrolysis of the ester substrates, pNPA or pNPB, progressively decreased as OP-hydrolase activity increased (Table 2). Thus, as OP-hydrolase activity is evolved to accommodate a pentavalent TS of an OP, the carboxylesterase activity and stabilization of a tetrahedral transition state is lost (Oppenoorth and van Asperen, 1960). For soman, the largest rate enhancements were observed (Table 5). Somanase activity was not observed in the G117H BChE single mutant (Millard et al., 1998) until a second mutation was added (G117H/E197Q). In pNBE, the A107H mutation (equivalent to G117H in BChE) enhanced the rate of spontaneous reactivation after soman inhibition, but an additional rate enhancement was achieved with the A107H/A190C variant. The *k*_r_ for A107H (*k*^Soman^_r_ = 0.7 ± 0.1 h^−1^) was 700-fold above WT (*k*^Soman^_r_ = 0.001 ± 0.004 h^−1^) and 4000-fold higher for the A107H/A190C variant (*k*^Soman^_r_ = 4 ± 1 h^−1^). The trends were similar to those observed with paraoxon (Table 4). A190 in pNBE is also at a different location than E197 in BChE, and rate enhancements in OP-hydrolase activity have not been reported from mutations at this site (Figure S1D).

**Table 4:** **Rates of reactivation after ethyl paraoxon inhibition measured for the DE variants at 37°C in 50 mM Tris pH 7.6, 150 mM NaCl, 2 mM BME**.

**Enzyme**	**CLONE**	*****k***_**reactivation**_ (1/h)**	**% Reactivation**
A107	D3	0.03 ± 0.01	110 ± 10
A107C	D4	0.15 ± 0.03	40 ± 3
A107D	D5	0.31 ± 0.02	90 ± 2
A107E	D6	0.048 ± 0.006	46 ± 4
A107F	D7	0.023 ± 0.004	130 ± 10
A107G	D8	0.0114 ± 0.0009	70 ± 4
A107H	–	0.53 ± 0.09	102 ± 5
A107I	D9	0.013 ± 0.004	70 ± 4
A107K	D10	0.04 ± 0.02	25 ± 6
A107L	D11	0.030 ± 0.005	25 ± 2
A107M	D12	0.06 ± 0.03	90 ± 10
A107N	E1	0.04 ± 0.01	60 ± 10
A107Q	E3	0.05 ± 0.02	110 ± 10
A107R	E4	0.14 ± 0.03	27 ± 2
A107S	E5	0.03 ± 0.01	100 ± 10
A107T	E6	0.034 ± 0.006	40 ± 5
A107V	E7	0.22 ± 0.03	28 ± 1
A107Y	E9	0.012 ± 0.003	7 ± 1
A107H/A190C	E10	1.5 ± 0.2^a^	62 ± 3
A107H/A190V	G2	0.4 ± 0.1	73 ± 9
A107H/A190G	F2	0.7 ± 0.3	90 ± 10
A107H/A190H	F3	0.10 ± 0.02	66 ± 8
A107H/A190M	F7	0.3 ± 0.2	17 ± 5
A107H/A400W	H10	0.4 ± 0.2	130 ± 50
A107H/A400M	H2	1.0 ± 0.2	97 ± 7
A107H/A400V	H9	0.6 ± 0.1	130 ± 20
A107H/A190C/A400T	A8	0.43 ± 0.07	92 ± 7
A107H/A190C/A400T	A8	1.0 ± 0.1^a^	75 ± 5
A107H/A190C/A400M	C4	1.0 ± 0.1^a^	75 ± 5

a Enzymes were heated at 37°C prior to reactivation.

**Table 5:** **Rates of reactivation after inhibition with soman**.

**Enzyme**	*****k***_**reactivation**_ (1/h)**	**% Reactivated**	**Fold increase**
WT	0.001 ± 0.004	<4% after 5.5 h	–
A107H	0.7 ± 0.1	106 ± 8	700
A107H/A190C^a^	1.8 ± 0.2	44 ± 5	1800
A107H/A190C^b^	4 ± 1	43 ± 6	4000
A107H/A190C/A400M^a^	0.7 ± 0.2	20 ± 2	700
A107H/A190C/A400M^b^	1.2 ± 0.5	17 ± 2	1200

a Without heating prior to inhibition.

b With 2 h of heating at 37°C prior to reactivation at 37°C.

The variant which displayed the greatest rate enhancements in OP-hydrolase activity, A107H/A190C, exhibited unexpected kinetic complexity consistent with a slow conformational change in the enzyme. Pre-incubation of the purified A107H/A190C enzyme at 37°C in the absence of any substrate or inhibitor caused a subsequent time-dependent increase in V_max_ for CE activity and the reactivation rate constants for selected OPAA (Figure S3). Maximal CE activity could be achieved by pre-incubating the enzyme at 37°C in 50 mM Tris pH 7.6, 150 mM NaCl, 2 mM BME for ≥2 h. Likewise, pre-equilibrating A107H/A190C to 37°C for ≥2 h doubled the apparent dephosphonylation rate constant following paraoxon or soman inhibition (Tables 4, 5). The dephosphorylation rate constant following DFP inhibition was not similarly affected. The DFP-inhibited A107H/A190C variant reactivated 5-fold more slowly than did A107H (Table 6), and no further increases could be gained by heating the enzyme. We also tested the triple mutant, A107H/A190C/A400M, for temperature-dependent hysteresis but found no significant effect on reactivation (Table 5).

**Table 6:** **Rates of reactivation at pH 7.6 after inhibition with DFP**.

**Enzyme**	*****k***_**reactivation**_ (1/h)**	**% Reactivated**
A107H	0.6 ± 0.1	110 ± 10
A107H/A190C	0.13 ± 0.08	150 ± 40
A107H/A190C^a^	0.17 ± 0.01	69 ± 2
A107H/A190G	0.63 ± 0.06	108 ± 3

a Heated for 3 h at 37°C prior to reactivation.

Several mutations at the A190 and A400 positions were compatible with A107H. The backbone NH groups of A107 and A190 form part of the oxyanion hole. Changes in the polarity of these NH groups have been proposed to enhance OPAAH activity (Yao et al., 2012). Hydrophobic mutations A400M and A400V within the loop slightly enhanced the rate of reactivation. The A107H/A400M (H2) and A107H/A190G (F2) double mutants showed the second largest enhancements, but additive effects were not observed in the A107H/A190C/A400M variant or any other triple mutant.

Having constructed a DE library with all 20 amino acids at position A107, we also determined if other residues at this position were more effective than histidine in catalyzing reactivation. In addition to A107H, the variants A107C, A107D, and A107V showed apparent reactivation rate enhancements for selected OPAA compared with WT pNBE. Of this group, however, only A107H and A107D fully reactivated after inhibition by paraoxon (Table 4). This result is similar to what was reported by Schopfer et al. (2004). Schopfer observed OP hydrolase activity in G117D, G117E, and L286H variants of BChE.

### Transfer of mutations onto hCE1

The spontaneous reactivation rate constant for WT hCE1 inhibited with paraoxon was low (Table 7). This is consistent with reports that WT hCE1 can be irreversibly inhibited by stereoisomers of soman or cyclosarin (Hemmert et al., 2010). The mutation equivalent to G117H in BChE was made in hCE1 (G143H), but did not enhance or confer OPAAH activity (Table 7). The hCE1 loop residues 302–320 (equivalent to 276–290 in BChE) that form the acyl pocket differ significantly among hCE1, pNBE, and BChE. In snake AChE, the single G122H mutation (homologous to BChE G117H) did not increase OPAAH activity; only introduction of two additional mutations (G122H/Y124Q/S125T) permitted engineering of limited spontaneous reactivation following slow inhibition with selected OPAA (Poyot et al., 2006). Thus, while pNBE is more similar to hCE1 in terms of substrate specificity, the utility of pNBE as a surrogate scaffold still remains to be explored.

**Table 7:** **Rates of reactivation of hCE1 after inhibition with paraoxon**.

**Enzyme**	**pH**	*****k***_**reactivation**_ (1/h)**	**% Reactivated**
hCE1 WT	7.0	0.078 ± 0.006	92 ± 3
	7.6	0.102 ± 0.006	98 ± 3
hCE1 G143H	7.0	0.025 ± 0.008	45 ± 8
	7.6	0.03 ± 0.03	15 ± 2
hCE1 G143H/A222C	7.0	0.007 ± 0.003	120 ± 60
	7.6	0.009 ± 0.007	11 ± 8

### Inhibition by paraoxon

Reliable measurement of IC_50_ or *K*_i_ values requires enzyme concentrations below the *K*_i_. For enzymes with IC_50_ values in the nM range, only upper limits can typically be measured. The minimum amount of enzyme needed to obtain a signal/noise ratio >2 was 0.5 nM of enzyme. The observed IC_50_ (0.37 nM) for paraoxon was almost equal with the enzyme concentration (0.5 nM), suggesting that the IC_50_ ≤ 0.5 nM. Thus, pNBE is an effective scavenger of paraoxon at low nM concentrations. Similar values have been reported for AChE with soman and sarin [IC^soman^_50_ = 0.88–2.53 nM, IC^sarin^_50_ = 3.27–6.15 nM (Fawcett et al., 2009)].

### Inhibition by echothiophate

pNBE and hCE1 share the cholinesterase fold, but lack cholinesterase activity. To determine if V-type inhibitors with choline-like leaving groups could be accommodated by variants, we screened the library with echothiophate and looked for irreversible inhibition. Through one mutation, A107S, we were able to achieve a 50-fold increase in the rate of inhibition. However, for the pNBE variants tested, the *K_p_* values remained high (millimolar range) compared with those of natural cholinesterases (Table 8).

**Table 8:** **Inhibition by echothiophate**.

**Enzyme**	*****k***_**2**_ (1/min)**	***K*_***p***_ (mM)**	***k***_**2**_/*K*_***p***_ (1/min*mM)
A107H	0.013 ± 0.005	9 ± 4	0.0014 ± 0.0008
A107K	0.014 ± 0.005	10 ± 4	0.0014 ± 0.0008
A107S	0.7 ± 0.4	10 ± 7	0.07 ± 0.06
A107T	0.06 ± 0.05	11 ± 8	0.006 ± 0.006
A107R	0.02 ± 0.04	>5	0.00045 ± 0.00009^a^
A107Q	0.079 ± 0.008	3 ± 1	0.026 ± 0.009
A107V	0.10 ± 0.02	20 ± 4	0.005 ± 0.001
A107Y	0.06 ± 0.04	20 ± 1	0.004 ± 0.004

Rates were measured using 1× Sorensen's buffer pH 7.4 at room temperature (22 ± 2°C).

a Inhibition was observed; however, the intercept could not be determined accurately from a distant extrapolation (very weak binding).

## Discussion

Arnold and colleagues have shown that *B. subtilis* pNBE can be modified to achieve increased thermostability, broadened substrate specificity, or improved reactivity in organic solvents using DE (Giver et al., 1998; Spiller et al., 1999; Brustad and Arnold, 2011). DE is a large scale site-directed mutagenesis experiment where selected residues are mutated to all 20 amino acids, or random mutations are introduced to alter catalytic activity and/or substrate specificity (Brustad and Arnold, 2011). This process generates 20 different enzymes for each selected site or thousands of variants with mutations at random sites (reviewed by Goldsmith and Tawfik, 2013); screening thousands of mutants is typically impractical. Several approaches are available for generating large libraries of mutants, but there are far fewer validated methods for selecting mutants with the desired activity. Here we constructed a “focused” DE library, utilized a bacterial homolog as a surrogate scaffold, and restricted the mutations to residues within a 7 Å radius of the nucleophilic serine. While pNBE, AChE, BChE, and hCE1 share a common fold (Figure S1), it is known that the single mutation analogous to G117H in BChE does not confer OP-hydrolase activity in AChE (Ordentlich et al., 1998; Poyot et al., 2006). Based upon substrate specificities, we show that pNBE and hCE1 are similar (this paper). However, when we examined the A107H variant of pNBE and the G123H variant of hCE1, we found that the histidine substitution only conferred OP-hydrolase activity in pNBE. Our preliminary results demonstrate that pNBE is a suitable prokaryotic scaffold for engineering improved reactivity with a range of OPAA inhibitors including soman, but that it is sufficiently different from hCE1 that additional mutations would be required.

While a significant enhancement in the rate of reactivation after soman inhibition was achieved (10^3^-fold increase, Table 5) the pNBE A107H variant did not achieve the same rates of reactivation as the BChE G117H variant [*k*^BChE−Soman^_r_ = 6000 ± 600 1/min (Millard et al., 1998) vs. *k*^pNBE−E10−Soman^_r_ = 0.07 ± 0.02 1/min]. This may in part be due to the more open active site of pNBE (Figure 2A) vs. the tunnel-like gorge of AChE and BChE.

One other complication was a slow time- and temperature-dependent change in activity in the variant which had the largest enhancement (10^3^-fold) in OP-hydrolase activity. Various forms of hysteresis in AChE and BChE have been observed kinetically (Masson et al., 2005; Badiou et al., 2008; Masson and Lockridge, 2010; Lushchekina et al., 2014), and possibly structurally (Nachon et al., 2011). Non-linear kinetic curves for BChE G117H also were observed with selected substrates (Millard et al., 1995b). Hysteresis affecting CE activity of both BChE and AChE (Masson et al., 2005; Badiou et al., 2008; Masson and Lockridge, 2010; Lushchekina et al., 2014) and OP-hydrolase activity (Masson, 2012) has been reported and has been attributed to the flipping of the His of the catalytic triad. A pronounced lag phase (3 min) was observed in the BChE A328C mutant at 25°C (Masson, 2012); the side chain of this residue is near His-438 of the triad (~4.5 Å). In pNBE the mechanism of hysteresis may or may not be the same since the A190 side chain is behind the oxyanion hole residues and is relatively distant from His-399 (>7 Å) (Figure S1). If the His of the catalytic triad is involved, however, the methionine residue in the A107H/A190C/A400M variant which did not display hysteresis may stabilize a particular rotamer of His-399. This mutant displayed a lower percentage of reactivated enzyme after soman inhibition when compared with A107H/A190C (Table 5) suggesting that conformational changes may be important in the mechanism of reactivation.

Hysteresis is rarely considered during DE screening, but can limit achievable rates of hydrolysis. It also complicates the interpretation of site-directed mutagenesis and structural studies since the crystallized structure may (or may not) represent the catalytically competent state. We observed kinetic complexity in the A107H/A190C pNBE variant that affected both esterase and OP-hydrolase activity. This suggests the involvement of a residue(s) which plays a role in both esterase and OP-hydrolase activity.

### Introduction of OPAAH activity to pNBE

The overarching goal of developing a nerve agent bioscavenger is to find or engineer a biocompatible enzyme that rapidly binds and hydrolyzes a broad range of neutral (G-type agents) and positively charged (V-type) OPAA under physiological conditions where the inhibitor is present at sub-micromolar concentrations. Cholinesterases react rapidly with all known OPAA nerve agents, but effectively remain inhibited irreversibly due to the stability of the OPAA-enzyme complex.

Introducing a single His (G117H) into human BChE converts the enzyme into a modest OPAAH by increasing the spontaneous reactivation rate constant while retaining reactivity with a broad range of inhibitors (Millard et al., 1995a; Lockridge et al., 1997). Follow-on attempts to incorporate His-117 into human or *Bungarus fasciatus* AChE were relatively unsuccessful (Poyot et al., 2006). pNBE is the second esterase to show an enhancement in OPAAH activity by introduction of a single His (A107H corresponds to G117H) and is significantly more amenable to *E. coli* expression.

Lockridge and colleagues rationally designed and tested more than 60 double or triple mutants of human BChE based upon the initial success with His-117, but none of these variants improved upon the OPAAH activity of G117H (Lockridge et al., 1997; Schopfer et al., 2004). We find a similar result using DE with pNBE. Although enhancements of spontaneous reactivation compared to WT were measured following paraoxon inhibition for pNBE A107D, A107V or A107C, the histidine mutant (A107H) showed the fastest and most complete dephosphorylation (Table 4). pNBE A107D is homologous with the blowfly CE G137D mutant that was isolated by screening OP-resistant populations of *Lucilia cuprina* for naturally occurring variants of G117H (Newcomb et al., 1997). A107D showed enhanced spontaneous reactivation compared with WT, but the turnover rates with paraoxon were slower than those of either pNBE A107H or the blowfly CE G137D (cf. Table 4 and Kirby et al., 2013).

Cholinesterases and carboxylesterases must stabilize a tetrahedral transition state to catalyze carboxyl ester hydrolysis, whereas the transition state of an organophosphate is generally a pentavalent trigonal bipyramid. Consequently, all attempts to engineer OPAAH activity into these enzymes must accept a significant risk of concomitant loss of natural esterase activity. Oppenoorth's “aliesterase hypothesis” was based upon this observed interchange in substrate specificities (Oppenoorth and van Asperen, 1960). Our results with pNBE generally confirmed this hypothesis with the trend showing that mutations increasing OPAAH activity also showed decreasing carboxylesterase activity (Tables 1–7).

The pNBE A107H/A190C variant showed a slow time- and temperature-dependent increase in CE activity and the rate of spontaneous reactivation following inhibition with paraoxon or soman (Figure S3; Tables 4, 5), but not with DFP (Table 6). DFP, unlike soman or paraoxon, has two bulky R-groups (Figure 1) which may restrict the pNBE active site from reaching the temperature-induced conformational change required for the higher level of activity. It has been shown that the DFP reaction significantly alters the conformation of the acyl pocket loop of AChE (Millard et al., 1999; Hornberg et al., 2007). The corresponding loop of pNBE is predicted to be nearby His-107 (Figure 2). Thus, the catalytically competent conformer of the histidine or hydrolytic water molecule may be affected by conformational changes in the loop. The simultaneous mutation of two residues (A107/A190) may permit subtle, local movements of the NH groups of the oxyanion hole that are sufficient to enhance catalysis (Yao et al., 2012). Alternatively, the double mutant may have more distal effects to structure the disordered loops of WT pNBE. It was shown previously that mutations which thermally stabilize the enzyme also increase the optimal temperature for pNBE carboxylesterase activity (Giver et al., 1998); the omega loop of the thermal stable pNBE variant (PDB 1C7I) is structured (Spiller et al., 1999).

### Importance of the oxyanion hole

Much of the catalytic power of serine hydrolases derives from the oxyanion hole (Bryan et al., 1986; Zhang et al., 2002; Warshel, 2003; Bobofchak et al., 2005), and we hypothesize that the same is true for engineered OPAAH activity. Millard and colleagues originally proposed the spontaneous reactivation of G117H was acid catalyzed and might involve a direct H-bond from the imidazolium to the phosphonyl (double bond) oxygen to stabilize the dephosphylation transition state, or an indirect steric effect that distorts the preformed electrostatic environment of the oxyanion hole and thereby permits the catalytic triad His-438 to catalyze reactivation (Millard et al., 1995a, 1998). Related and alternative mechanisms subsequently have been proposed (Lockridge et al., 1997; Newcomb et al., 1997; Albaret et al., 1998; Schopfer et al., 2004; Poyot et al., 2006; Nachon et al., 2011; Yao et al., 2012), supported, or refuted based upon analogy with follow-on His-117 mutations to related enzymes, molecular modeling studies (Amitay and Shurki, 2009; Yao et al., 2012) or static, medium resolution X-ray crystal structures (Masson et al., 2007); however, the actual enzyme mechanism of G117H remains unresolved.

Our studies on the structurally homologous pNBE mutants may provide useful data for ongoing efforts to elucidate the G117H mechanism. First, like G117H, placing a histidine residue at the homologous A107H position in the oxyanion hole enhanced OPAAH activity with a range of inhibitors (Tables 4, 5). Second, OPAAH activity increased as the pH decreased from 7.6 to 7.0, consistent with a mechanism that is acid-catalyzed. Third, the A190C mutation further enhanced the rate of reactivation of the A107H mutation. The NH group of A190 forms part of the 3-point oxyanion hole, and the side chain would be expected to point away from the oxyanion. Finally, we observed a slow time- and temperature-dependent change in carboxylesterase and OPAAH activity of the A107H/A190C variant that may be consistent with a conformational change or some other reversible modification in the free enzyme which enhances the role of these residues in catalysis. Additional work is required to determine if these observations can be translated to improve human BChE G117H activity.

### Introduction of limited cholinesterase activity

One objective of this work was to determine if cholinesterase activity could be introduced into pNBE. The active site cavity of pNBE is formed by four loops that are largely disordered in the WT enzyme crystal structure, viz. residues 64–71 (unstructured) and 413–417 (unstructured) on one side of the active site, and 316–320 (unstructured) and 260–268 (structured) on the other side (Spiller et al., 1999). It appears that these flexible loops become longer, more differentiated and ordered through evolution to form the substrate specificity loops observed in the X-ray structures of AChE and BChE. One side becomes the cholinesterase “acyl pocket loop,” which we have shown previously to have reversible conformational flexibility in *Torpedo californica* (*Tc*) AChE when binding selected OPAA (Millard et al., 1999; Hornberg et al., 2007). The other side develops the so-called Ω-loop carrying Trp-84 (*Tc*AChE numbering; Trp-82 in BChE), a residue that complements trimethyl or choline-like substrate leaving groups.

Residues corresponding to the cholinesterase Ω-loop are disordered in the structure of WT pNBE [PDB 1QE3 (Spiller et al., 1999)]. Both pNBE and hCE1 lack the critical Trp-84 side chain (Figure 2E) (Satoh and Hosokawa, 1995; Imai et al., 2006), and this probably explains why these enzymes are relatively poor at binding cationic substrates (e.g., ATCh and BTCh; Table 3) or echothiophate (Table 8). Our initial experiment to insert the entire Ω-loop into pNBE is a first step in evolving the bacterial enzyme toward a cholinesterase. The loop was accepted by the pNBE fold and had little or no effect on the reactivity of the enzyme with neutral substrates or inhibitor; however it also did not result in detectable activity with positively charged substrates (Table 3). While this rational design attempt with the Ω-loop failed to increase cholinesterase activity, the focused DE exploration succeeded in finding A107K which demonstrated an almost 10-fold increase in its specificity constant for benzyolthiocholine compared with WT.

### Comparison of pNBE and hCE1

Human carboxylesterase has been proposed as an alternative or adjunct bioscavenger to the cholinesterases because hCE1 is abundant in human liver, binds and hydrolyzes some neutral OPAA nerve agents, and does not undergo significant aging after inhibition with the most deadly OPAA nerve agent, soman (Hemmert et al., 2010). However, the primary limitation to using hCE as a nerve agent bioscavenger is the slow reaction rates with positively charged OPAA. In our study, cholinesterase activity could be introduced into pNBE by the A107K mutation, but the amount was still several orders of magnitude below that of cholinesterases and the mutation had no effect on the bimolecular rate constant for inhibition by a cationic OPAA (echothiophate; Table 8). More importantly, the G143H mutation did not confer OPAAH activity in hCE1 (Table 7).

In summary, along with its primary sequence and structural homology to the cholinesterases and the shared use of a rare Glu residue instead of Asp in the catalytic triad, we have shown that *B. subtilis* pNBE can accommodate the cholinesterase Ω-loop without detriment to protein folding or endogenous esterase activity. We have also identified an unexpected point mutation (A107K) that significantly increases turnover of a positively charged substrate. Moreover, like BChE but not AChE or hCE1, the pNBE structure accepts substitutions (A107H or A107H/A190C) corresponding with G117H that confer significant OPAAH activity, thereby expanding the enzyme's natural substrate specificity to include phosphoric and phosphonic acid esters. Taken together, these results suggest to us that pNBE is an excellent prokaryotic scaffold for follow-on DE studies, as well as other methods like incorporation of unnatural amino acids, that can inform new pathways for continued engineering of useful cholinesterase and/or OPAAH activity within the α/β-hydrolase superfamily.

### Conflict of interest statement

While the Guest Associate Editor Carissa M. Soto and the author Patricia. M. Legler are affiliated to the same Institution, the review process was handled objectively as established by the journal guidelines. The authors declare that the research was conducted in the absence of any commercial or financial relationships that could be construed as a potential conflict of interest.

## Supplementary material

A graph of the esterase activities of the individual mutants in the DE library, evidence of reversible hysteresis, and sequence alignments of the enzymes discussed in this study are included in the Supplemental Information.

Scheme S1 **Organophosphate inhibition, spontaneous reactivation, and aging**.

Figure S1 **Acylation-the first step in the catalytic cycle. (A)** The serine hydrolases AChE (2ACE), BChE (1P0M), hCE1 (2HRR), and pNB-esterase (1QE3) all share a common fold, but have very different substrate specificities. This is in part due to residues within the Ω-loop (colored red) which form part of the choline binding pocket in AChe and BChE. The equivalent loop in pNBE is disordered in the crystal structure 1QE3. **(B)** The acylation step and active site of pNBE. Residues of the catalytic triad are boxed. **(C)** The acylation step and active site of BChE. The cationic choline ester is accommodated by Glu-197 and Trp-82. Trp-82 of the Ω-loop makes an important cation-pi interaction with the choline ester and V-type agents (VX and VR) which mimic choline esters. **(D)** Alternate views of the Ala-190 side chain. A190 is behind the loop of the oxyanion hole residues G106 and A107.

Figure S2 **Specific activities (S.A., U/mg) for all 95 variants in the DE library using five substrates: pNPA, pNPB, benzoylthiocholine, butyrylthiocholine, and acetylthiocholine**. S.A. from single point assays were measured using 5, 1, 2.5, 2.5, and 2.5 mM of the substrates, respectively. In well D3 is the WT pNBesterase enzyme (where A107 is an alanine). For all other mutants the A107H mutation is present in combination with the denoted mutation. For the A107X mutants (where X = any amino acid), only the single mutation is present. Note, the y-scale has been adjusted for some graphs. For variants with no visible bars, values near the spontaneous rate of hydrolysis were measured and plotted. One substrate, pNPB, was hydrolyzed at detectable levels for all of the mutants. The S.A. were needed to determine the number of Units per well to use in the microscale reactivation rate experiments described. Steady state kinetic parameters were measured for selected variants which showed enhancements in esterase activity and are described in Tables 2, 3. Reductions in carboxylesterase activity were expected for variants which had acquired OP-hydrolase activity [Aliesterase Hypothesis (Oppenoorth and van Asperen, 1960)]. SDS-PAGE gel showing the purity of 10 variants purified using small scale preps and single columns.

Figure S3 **Effect of hysteresis on carboxylester hydrolysis**. The E10 A107H/A190C double variant showed a 6-fold increase in its carboxylesterase activity after elution from a PD-10 column during 37°C incubation periods. For assays, 10 μL of the enzyme was added to a 1 mL reaction volume, and rates were measured at R.T. This dramatic increase in activity was not observed for any other variant.

Supplemental Information **Sequence alignment of the constructs discussed**. The pNBE sequence corresponds to P37967; it contains strain-specific differences from the sequence of PDB 1QE3.

## Abbreviations

AtCh: acetylthiocholine
BME: beta-mercaptoethanol
BtCh: butyrylthiocholine
BzCh: benzoylthiocholine
CD: circular dichroism
CE: carboxylesterase
DMSO: dimethylsulfoxide
DTNB: dithiobis(2-nitrobenzoic acid)
DTT: dithiothreitol
EB: equilibration buffer
hCE1: human carboxylesterase 1
IPTG: isopropyl-β-thiogalactoside
Ω-loop: residues between Cys-67-Cys-94 (TcAChE numbering)
OPAA: organophosphorus acid anhydride inhibitors
OPAAH: organophosphorus acid anhydride hydrolase
paraoxon: diethyl *p*-nitrophenylphosphate
pNBE: p-nitrobenzylesterase
pNPA: p-nitrophenyl acetate
pNPB: p-nitrophenyl butyrate
SDS-PAGE: sodium dodecyl sulfate polyacrylamide gel electrophoresis
WT: wild type.
