# Supplementary figures and images for: Development of organophosphate hydrolase activity in a bacterial homolog of human cholinesterase
Source: Front Chem. 2014 Jul 16;2:46. doi: 10.3389/fchem.2014.00046 (PMC4100338; doi:10.3389/fchem.2014.00046)

**Scheme S1.** Organophosphate Inhibition, Spontaneous Reactivation, and Aging.

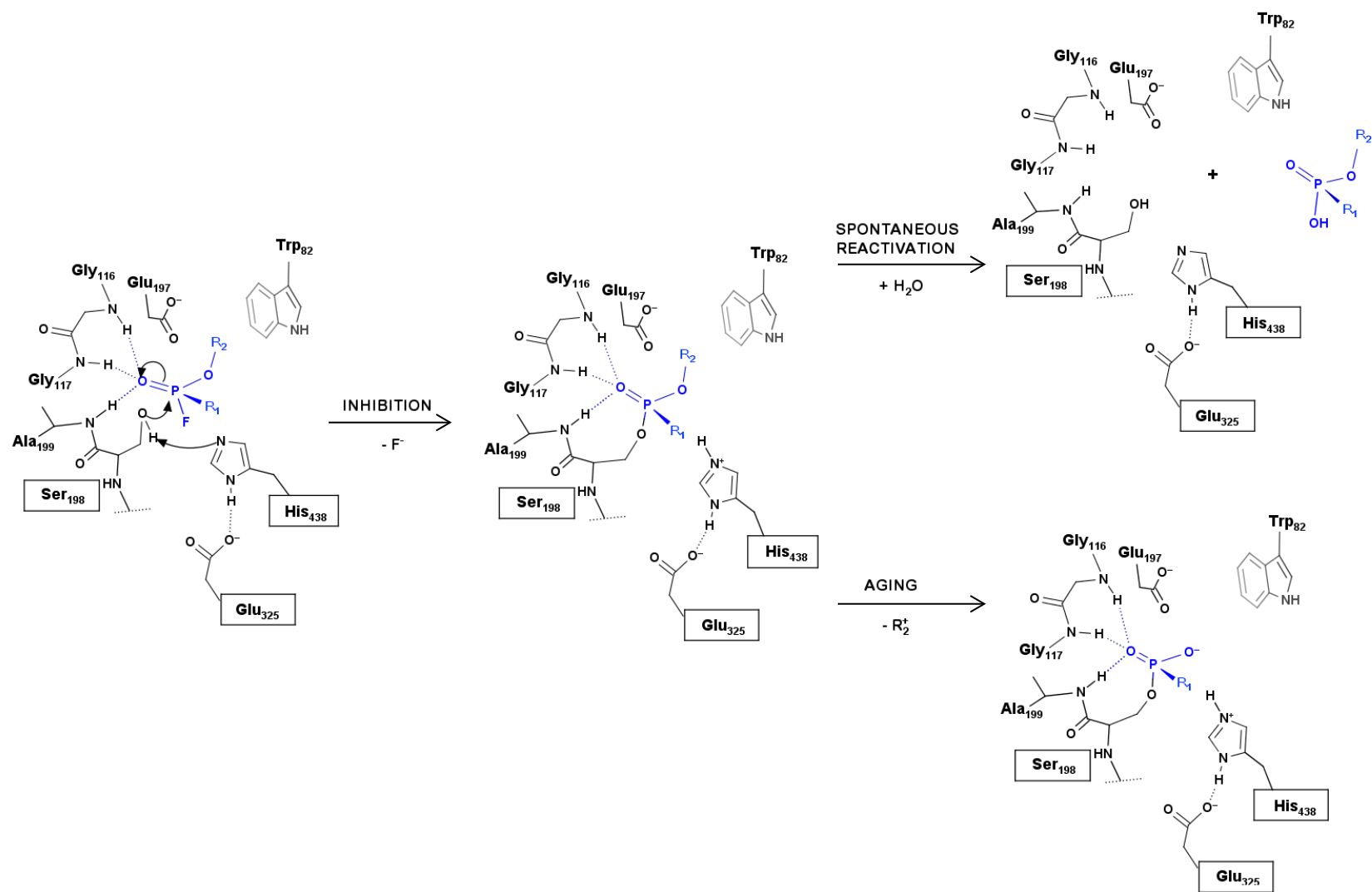

Supplement: Scheme S1 — Organophosphate inhibition, spontaneous reactivation, and aging. [file DataSheet1.PDF]
