# Supplementary material for: Development of organophosphate hydrolase activity in a bacterial homolog of human cholinesterase
Source: Front Chem. 2014 Jul 16;2:46. doi: 10.3389/fchem.2014.00046 (PMC4100338; doi:10.3389/fchem.2014.00046)
Supplement: Figure S1 — Acylation-the first step in the catalytic cycle. (A) The serine hydrolases AChE (2ACE), BChE (1P0M), hCE1 (2HRR), and pNB-esterase (1QE3) all share a common fold, but have very different substrate specificities. This is in part due to residues within the Ω-loop (colored red) which form part of the choline binding pocket in AChe and BChE. The equivalent loop in pNBE is disordered in the crystal structure 1QE3. (B) The acylation step and active site of pNBE. Residues of the catalytic triad are boxed. (C) The acylation step and active site of BChE. The cationic choline ester is accommodated by Glu-197 and Trp-82. Trp-82 of the Ω-loop makes an important cation-pi interaction with the choline ester and V-type agents (VX and VR) which mimic choline esters. (D) Alternate views of the Ala-190 side chain. A190 is behind the loop of the oxyanion hole residues G106 and A107. [file DataSheet2.DOCX]

**Suppl. Fig. S1. Acylation –the first step in the catalytic cycle. (A)** The serine hydrolases AChE (2ACE), BChE (1P0M), hCE1 (2HRR) and pNB-esterase (1QE3) all share a common fold, but have very different substrate specificities. This is in part due to residues within the Ω-loop (colored red) which form part of the choline binding pocket in AChe and BChE. The equivalent loop in pNBE is disordered in the crystal structure 1QE3. **(B)** The acylation step and active site of pNBE. Residues of the catalytic triad are boxed. **(C)** The acylation step and active site of BChE. The cationic choline ester is accommodated by Glu-197 and Trp-82. Trp-82 of the Ω-loop makes an important cation-pi interaction with the choline ester and V-type agents (VX and VR) which mimic choline esters. (D) Alternate views of the Ala-190 side chain. A190 is behind the loop of the oxyanion hole residues G106 and A107.


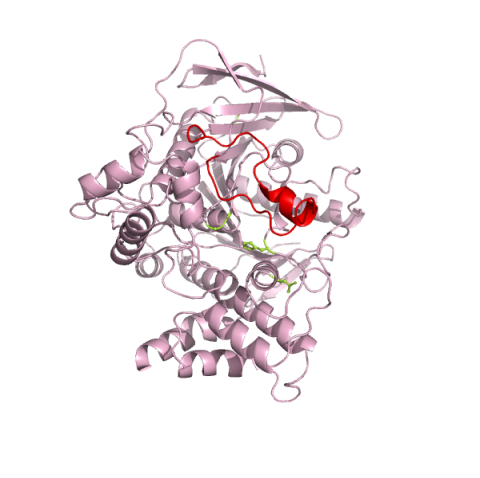

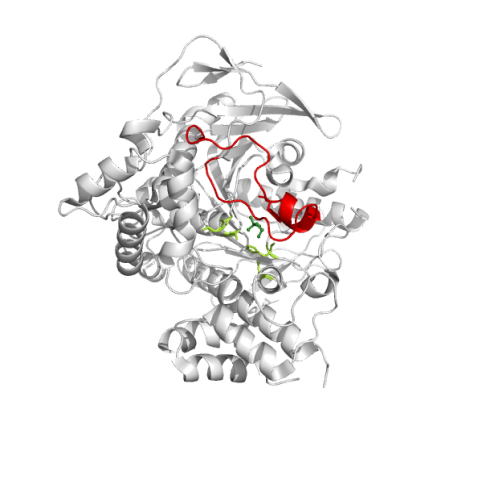

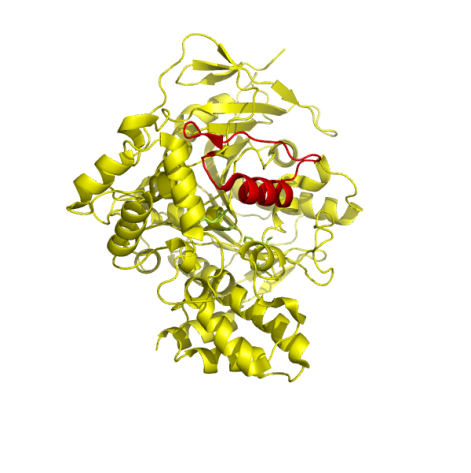

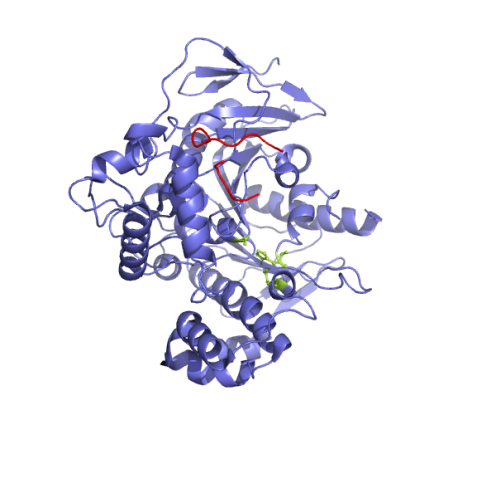


**A**

TcAChE BChE hCE1 pNBE

**B**


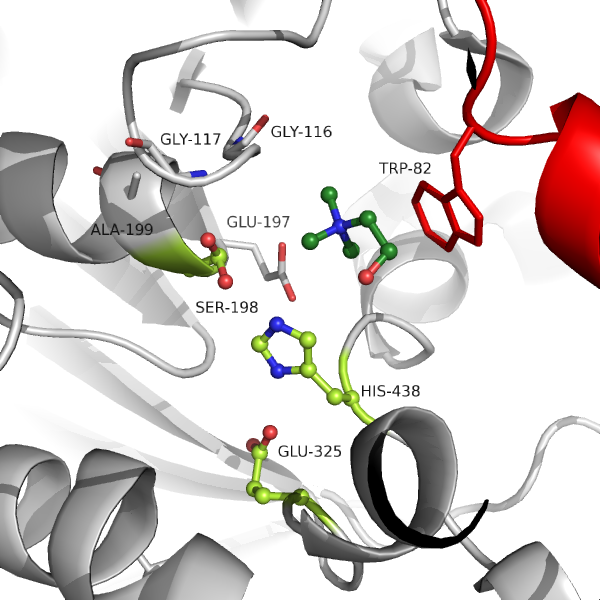

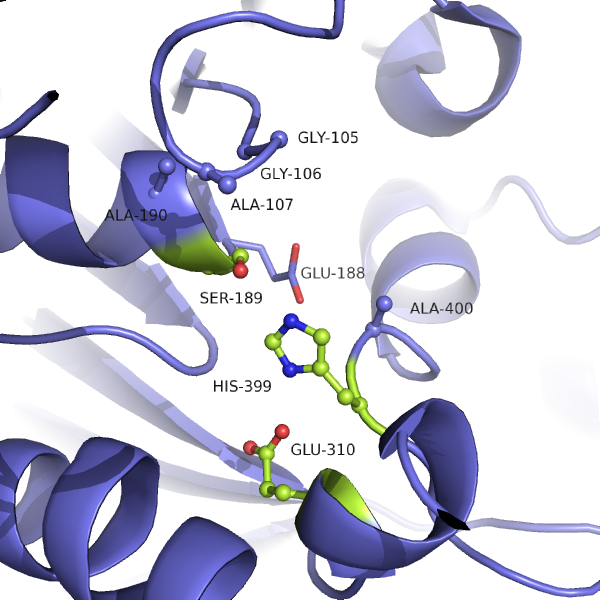

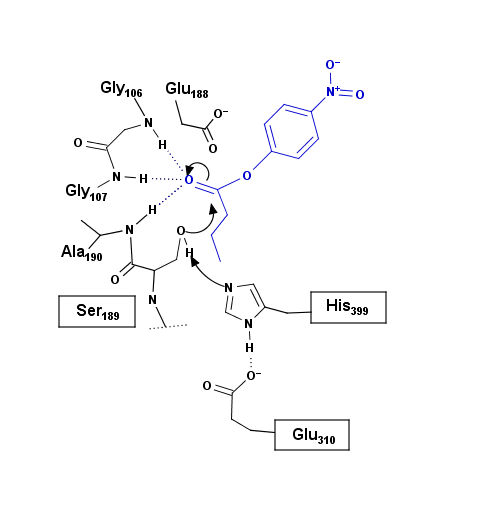


**
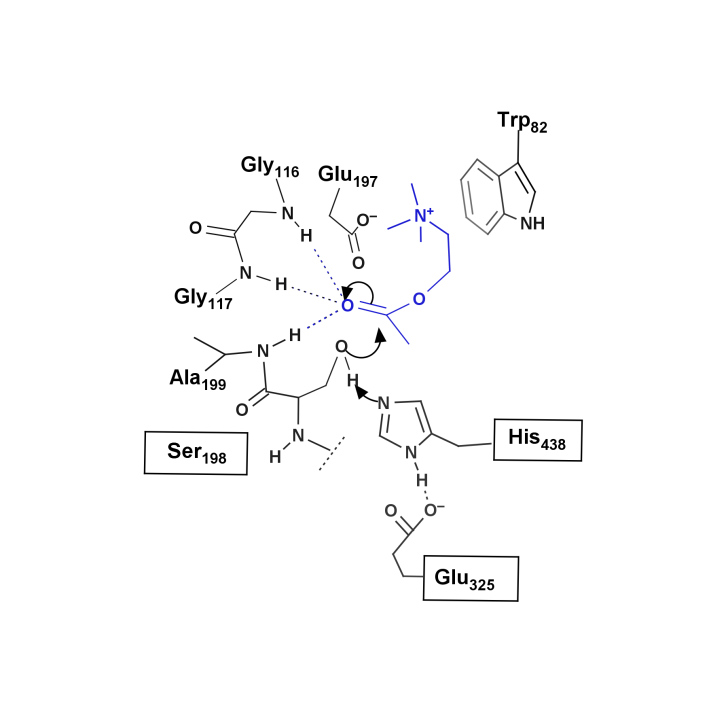
C**

**D**


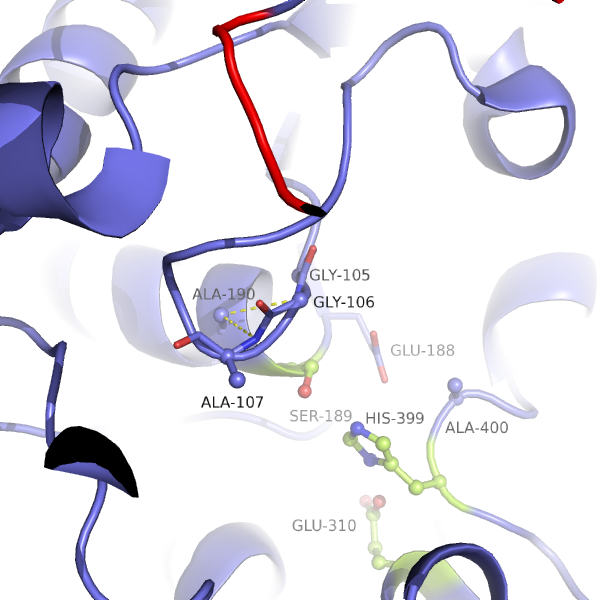

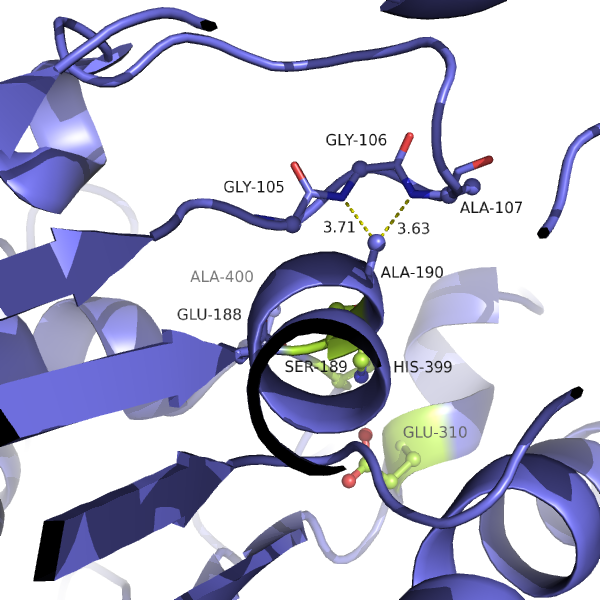


**Back**

**Front**

***Oxyanion***

***Hole***

**Suppl. Fig. S2**. Specific activities (S.A., U/mg) for all 95 variants in the DE library using five substrates: pNPA, pNPB, benzoylthiocholine, butyrylthiocholine, and acetylthiocholine. S.A. from single point assays were measured using 5 mM, 1 mM, 2.5 mM, 2.5 mM, and 2.5 mM of the substrates, respectively. In well D3 is the WT pNBesterase enzyme (where A107 is an alanine). For all other mutants the A107H mutation is present in combination with the denoted mutation. For the A107X mutants (where X = any amino acid), only the single mutation is present. Note, the y-scale has been adjusted for some graphs. For variants with no visible bars, values near the spontaneous rate of hydrolysis were measured and plotted. One substrate, pNPB, was hydrolyzed at detectable levels for all of the mutants. The S.A. were needed to determine the number of Units per well to use in the microscale reactivation rate experiments described. Steady state kinetic parameters were measured for selected variants which showed enhancements in esterase activity and are described in **Tables 2** and **3**. Reductions in carboxylesterase activity were expected for variants which had acquired OP-hydrolase activity (Aliesterase Hypothesis (*62*)).

**Suppl. Fig. S2. Continued.** SDS-PAGE gel showing the purity of 10 variants purified using small scale preps and single columns.

M A12 B5 B7 B10 C6 D3 D4 D5 D7 D8 D9


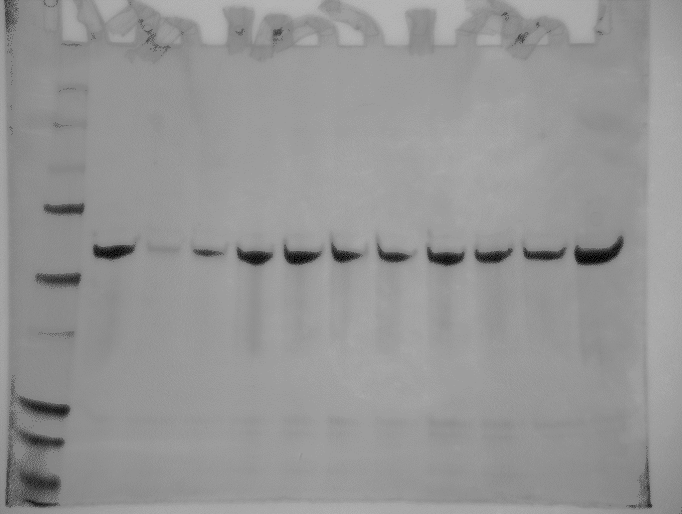


**Suppl. Fig. S3. Effect of hysteresis on carboxylester hydrolysis.** The E10 A107H/A190C double variant showed a 6-fold increase in its carboxylesterase activity after elution from a PD-10 column during 37 °C incubation periods. For assays, 10 μL of the enzyme was added to a 1 mL reaction volume, and rates were measured at R.T. This dramatic increase in activity was not observed for any other variant.

**Supplemental Information.** Sequence alignment of the constructs discussed. The pNBE sequence corresponds to P37967; it contains strain-specific differences from the sequence of PDB 1QE3.

CLUSTAL 2.1 multiple sequence alignment

P37967 -----------------------------MTHQIVTTQYGKVKG------TTENGVHKWK 25

pNB_WT MAISRELVDPMGSSHHHHHHSSGLEVLFQGPHQIVTTQYGKVKG------TTENGVHKWK 54

BChE_pNB A107H MAISRELVDPMGSSHHHHHHSSGLEVLFQGPHQIVTTQYGKVKG------TTENGVHKWK 54

1QE3_pNB -----------------------------MTHQIVTTQYGKVKG------TTENGVHKWK 25

4BDS_BChE -----------------------------EDDIIIATKNGKVRGMQL--TVFGGTVTAFL 29

2XMB_BChEG117H -----------------------------EDDIIIATKNGKVRGMQL--TVFGGTVTAFL 29

2ACE_TcAChE ---------------------------DDHSELLVNTKSGKVMGTRV--PVLSSHISAFL 31

2HRR_HCE1 -----------------------------SSPPVVDTVHGKVLGKFVSLEGFAQPVAIFL 31

:: * *** * : :

P37967 GIPYAKPPVGQWRFKAPEPPEVWEDVLDATAYGSICPQ--------PSDLLSLSYTELPR 77

pNB_WT GIPYAKPPVGQWRFKAPEPPEVWEDVLDATAYGSICPQ--------PSDLLSLSYTELPR 106

BChE_pNB A107H GIPYAKPPVGQWRFKAPEPPEVWEDVLDATAYGSICCQN--IDQSFPGFHGSEMWNPNTD 112

1QE3_pNB GIPYAKPPVGQWRFKAPEPPEVWEDVLDATAYGPICPQ--------PSDLLSLSYTELPR 77

4BDS_BChE GIPYAQPPLGRLRFKKPQSLTKWSDIWNATKYANSCCQN--IDQSFPGFHGSEMWNPNTD 87

2XMB_BChEG117H GIPYAQPPLGRLRFKKPQSLTKWSDIWNATKYANSCCQN--IDQSFPGFHGSEMWNPNTD 87

2ACE_TcAChE GIPFAEPPVGNMRFRRPEPKKPWSGVWNASTYPNNCQQY--VDEQFPGFSGSEMWNPNRE 89

2HRR_HCE1 GIPFAKPPLGPLRFTPPQPAEPWSFVKNATSYPPMCTQDPKAGQLLSELFTNRKENIPLK 91

***:*:**:* ** *:. *. : :*: * * * . . .

P37967 QSEDCLYVNVFAPDTPSKN--LPVMVWIHGGAFYLGAGSEPLYDGSKLAAQGEVIVVTLN 135

pNB_WT QSEDCLYVNVFAPDTPSKN--LPVMVWIHGGAFYLGAGSEPLYDGSKLAAQGEVIVVTLN 164

BChE_pNB A107H LSEDCLYVNVFAPDTPSKN--LPVMVWIHGGHFYLGAGSEPLYDGSKLAAQGEVIVVTLN 170

1QE3_pNB QSEDCLYVNVFAPDTPSQN--LPVMVWIHGGAFYLGAGSEPLYDGSKLAAQGEVIVVTLN 135

4BDS_BChE LSEDCLYLNVWIPAPKPKN--ATVLIWIYGGGFQTGTSSLHVYDGKFLARVERVIVVSMN 145

2XMB_BChEG117H LSEDCLYLNVWIPAPKPKN--ATVLIWIYGGHFQTGTSSLHVYDGKFLARVERVIVVSMN 145

2ACE_TcAChE MSEDCLYLNIWVPSPRPKS--TTVMVWIYGGGFYSGSSTLDVYNGKYLAYTEEVVLVSLS 147

2HRR_HCE1 LSEDCLYLNIYTPADLTKKNRLPVMVWIHGGGLMVGAAS--TYDGLALAAHENVVVVTIQ 149

******:*:: * .:. .*::**:** : *:.: *:* ** .*::*::.

P37967 YRLGPFGFLHLSSFNEAYSDNLGLLDQAAALKWVRENISAFGGDPDNVTVFGESAGGMSI 195

pNB_WT YRLGPFGFLHLSSFNEAYSDNLGLLDQAAALKWVRENISAFGGDPDNVTVFGESAGGMSI 224

BChE_pNB A107H YRLGPFGFLHLSSFNEAYSDNLGLLDQAAALKWVRENISAFGGDPDNVTVFGESAGGMSI 230

1QE3_pNB YRLGPFGFLHLSSFDEAYSDNLGLLDQAAALKWVRENISAFGGDPDNVTVFGESAGGMSI 195

4BDS_BChE YRVGALGFLALPGNPEAPGN-MGLFDQQLALQWVQKNIAAFGGNPKSVTLFGESAGAASV 204

2XMB_BChEG117H YRVGALGFLALPGNPEAPGN-MGLFDQQLALQWVQKNIAAFGGNPKSVTLFGESAGAASV 204

2ACE_TcAChE YRVGAFGFLALHGSQEAPGN-VGLLDQRMALQWVHDNIQFFGGDPKTVTIFGESAGGASV 206

2HRR_HCE1 YRLGIWGFFSTG--DEHSRGNWGHLDQVAALRWVQDNIASFGGNPGSVTIFGESAGGESV 207

**:* **: * . * :** **:**:.** ***:* .**:******. *:

P37967 AALLAMPAAKGLFQKAIMESG------ASRTMTKEQAASTSAAFLQVLGINEGQLDKLHT 249

pNB_WT AALLAMPAAKGLFQKAIMESG------ASRTMTKEQAASTSAAFLQVLGINEGQLDKLHT 278

BChE_pNB A107H AALLAMPAAKGLFQKAIMESG------ASRTMTKEQAASTSAAFLQVLGINEGQLDKLHT 284

1QE3_pNB AALLAMPAAKGLFQKAIMESG------ASRTMTKEQAASTAAAFLQVLGINESQLDRLHT 249

4BDS_BChE SLHLLSPGSHSLFTRAILQSGSFNAPWAVTSLYEARNRTLNLAKLTGCSRENETEIIKCL 264

2XMB_BChEG117H SLHLLSPGSHSLFTRAILQSGSFNAPWAVTSLYEARNRTLNLAKLTGCSRENETEIIKCL 264

2ACE_TcAChE GMHILSPGSRDLFRRAILQSGSPNCPWASVSVAEGRRRAVELGRNLNCNLNSDEELIHCL 266

2HRR_HCE1 SVLVLSPLAKNLFHRAISESG-VALTSVLVKKGDVKPLAEQIAITAGCKTTTSAVMVHCL 266

. : * ::.** :** :** . . . : : .

P37967 VSAEDLLKAADQLRIAEKENIFQ-------LFFQPALDPKTLPEEPEKAIAEGAASGIPL 302

pNB_WT VSAEDLLKAADQLRIAEKENIFQ-------LFFQPALDPKTLPEEPEKAIAEGAASGIPL 331

BChE_pNB A107H VSAEDLLKAADQLRIAEKENIFQ-------LFFQPALDPKTLPEEPEKAIAEGAASGIPL 337

1QE3_pNB VAAEDLLKAADQLRIAEKENIFQ-------LFFQPALDPKTLPEEPEKSIAEGAASGIPL 302

4BDS_BChE RNKDPQEILLNEAFVVPYGTPLS-------VNFGPTVDGDFLTDMPDILLELGQFKKTQI 317

2XMB_BChEG117H RNKDPQEILLNEAFVVPYGTPLS-------VNFGPTVDGDFLTDMPDILLELGQFKKTQI 317

2ACE_TcAChE REKKPQELIDVEWNVLPFDSIFR-------FSFVPVIDGEFFPTSLESMLNSGNFKKTQI 319

2HRR_HCE1 RQKTEEELLETTLKMKFLSLDLQGDPRESQPLLGTVIDGMLLLKTPEELQAERNFHTVPY 326

: : : ..:* : :

P37967 LIGTTRDEG----YLFFTPDSDVHSQETLDAALEYLLGKPLAEKVADLYPRSLESQIHMM 358

pNB_WT LIGTTRDEG----YLFFTPDSDVHSQETLDAALEYLLGKPLAEKVADLYPRSLESQIHMM 387

BChE_pNB A107H LIGTTRDEG----YLFFTPDSDVHSQETLDAALEYLLGKPLAEKVADLYPRSLESQIHMM 393

1QE3_pNB LIGTTRDEG----YLFFTPDSDVHSQETLDAALEYLLGKPLAEKAADLYPRSLESQIHMM 358

4BDS_BChE LVGVNKDEG----TAFLVYGAPGFSKDNNSIITRKEFQEGLKIFFPGVSEFGKESILFHY 373

2XMB_BChEG117H LVGVNKDEG----TAFLVYGAPGFSKDNNSIITRKEFQEGLKIFFPGVSEFGKESILFHY 373

2ACE_TcAChE LLGVNKDEG----SFFLLYGAPGFSKDSESKISREDFMSGVKLSVPHANDLGLDAVTLQY 375

2HRR_HCE1 MVGINKQEFGWLIPMLMSYPLSEGQLDQKTAMSLLWKSYPLVCIAKELIPEATEKYLGGT 386

::* .::* :: . : : . :

P37967 TDLL--------------------FWRPAVAYASAQSHYAP-VWMYRFDWHPK-----KP 392

pNB_WT TDLL--------------------FWRPAVAYASAQSHYAP-VWMYRFDWHPK-----KP 421

BChE_pNB A107H TDLL--------------------FWRPAVAYASAQSHYAP-VWMYRFDWHPK-----KP 427

1QE3_pNB TDLL--------------------FWRPAVAYASAQSHYAP-VWMYRFDWHPE-----KP 392

4BDS_BChE TDWVDDQRPENYREALGDVVGDYNFICPALEFTKKFSEWGNNAFFYYFEHRSSKL--PWP 431

2XMB_BChEG117H TDWVDDQRPENYREALGDVVGDYNFICPALEFTKKFSEWGNNAFFYYFEHRSSKL--PWP 431

2ACE_TcAChE TDWMDDNNGIKNRDGLDDIVGDHNVICPLMHFVNKYTKFGNGTYLYFFNHRASNL--VWP 433

2HRR_HCE1 DDTVKK------KDLFLDLIADVMFGVPSVIVARNHRDAGAPTYMYEFQYRPSFSSDMKP 440

* : . * : . . . .::* *: :.. *

P37967 PYNKAFHALELPFVFGNLDGLERMAKAEITDEVKQLSHTIQSAWITFAKTGNPS---TEA 449

pNB_WT PYNKAFHALELPFVFGNLDGLERMAKAEITDEVKQLSHTIQSAWITFAKTGNPS---TEA 478

BChE_pNB A107H PYNKAFHALELPFVFGNLDGLERMAKAEITDEVKQLSHTIQSAWITFAKTGNPS---TEA 484

1QE3_pNB PYNKAFHALELPFVFGNLDGLERMAKAEITDEVKQLSHTIQSAWITFAKTGNPS---TEA 449

4BDS_BChE EWMGVMHGYEIEFVFG----LPLERRDQYTKAEEILSRSIVKRWANFAKYGNPQETQNQS 487

2XMB_BChEG117H EWMGVMHGYEIEFVFG----LPLERRDQYTKAEEILSRSIVKRWANFAKYGNPQETQNQS 487

2ACE_TcAChE EWMGVIHGYEIEFVFG----LPLVKELNYTAEEEALSRRIMHYWATFAKTGNPNEPHSQE 489

2HRR_HCE1 KTVIGDHGDELFSVFG-----APFLKEGASEEEIRLSKMVMKFWANFARNGNPNG--EGL 493

*. *: *** . : **: : * .**: ***.

P37967 VNWPAYHEETRETLILDSEITIENDPESEKRQKLFPSKGE-------- 489

pNB_WT VNWPAYHEETRETLILDSEITIENDPESEKRQKLFPSKGE-------- 518

BChE_pNB A107H VNWPAYHEETRETLILDSEITIENDPESEKRQKLFPSKGE-------- 524

1QE3_pNB VNWPAYHEETRETVILDSEITIENDPESEKRQKLFPSKGE-------- 489

4BDS_BChE TSWPVFKSTEQKYLTLNTESTRIMTKLRAQQCRFWTSFFPKV------ 529

2XMB_BChEG117H TSWPVFKSTEQKYLTLNTESTRIMTKLRAQQCRFWTSFFPKV------ 529

2ACE_TcAChE SKWPLFTTKEQKFIDLNTEPMKVHQRLRVQMCVFWNQFLPKLLNATAC 537

2HRR_HCE1 PHWPEYNQKEG-YLQIG-ANTQAAQKLKDKEVAFWTNLFAK------- 532

** : : :. : :: .
